# An epigenetic regulator-related score (EpiScore) predicts survival in patients with diffuse large B cell lymphoma and identifies patients who may benefit from epigenetic therapy

**DOI:** 10.18632/oncotarget.24901

**Published:** 2018-04-10

**Authors:** Vanessa Szablewski, Caroline Bret, Alboukadel Kassambara, Julie Devin, Guillaume Cartron, Valérie Costes-Martineau, Jérôme Moreaux

**Affiliations:** ^1^ Department of Biological Hematology, CHU Montpellier, Montpellier, France; ^2^ Institute of Human Genetics, CNRS-UM UMR 9002, Montpellier, France; ^3^ University of Montpellier, UFR de Médecine, Montpellier, France; ^4^ Department of Biopathology, CHU Montpellier, Montpellier, France; ^5^ CHU Montpellier, Department of Clinical Hematology, Montpellier, France; ^6^ Montpellier University, UMR CNRS 5235, Montpellier, France

**Keywords:** epigenetics, Diffuse large B cells lymphoma, prognostic value, gene expression profiling, targeted treatment

## Abstract

Diffuse large B-cell lymphoma (DLBCL) is the most common form of lymphoma and shows considerable clinical and biological heterogeneity. Much research is currently focused on the identification of prognostic markers for more specific patients’ risk stratification and on the development of therapeutic approaches to improve the long-term outcome. Epigenetic alterations are involved in various cancers, including lymphoma. Interestingly, epigenetic alterations are reversible and drugs to target some of them have been developed. In this study, we demonstrated that the gene expression profile of epigenetic regulators has a prognostic value in DLBCL and identified pathways that could be involved in DLBCL poor outcome. We then designed a new risk score (EpiScore) based on the gene expression level of the epigenetic regulators DNMT3A, DOT1L, SETD8. EpiScore was predictive of overall survival in DLBCL and allowed splitting patients with DLBCL from two independent cohorts (*n =* 414 and *n =* 69) in three groups (high, intermediate and low risk). EpiScore was an independent predictor of survival when compared with previously described prognostic factors, such as the International Prognostic Index (IPI), germinal center B cell and activated B cell molecular subgroups, gene expression-based risk score (GERS) and DNA repair score. Immunohistochemistry analysis of DNMT3A in 31 DLBCL samples showed that DNMT3A overexpression (>42% of positive tumor cells) correlated with reduced overall and event-free survival. Finally, an HDAC gene signature was significantly enriched in the DLBCL samples included in the EpiScore high-risk group. We conclude that EpiScore identifies high-risk patients with DLBCL who could benefit from epigenetic therapy.

## INTRODUCTION

Diffuse large B-cell lymphoma (DLBCL) is the most common lymphoma type and accounts for 30–40% of newly diagnosed non-Hodgkin lymphoma (NHL) cases in adults [1]. DLBCL is a heterogeneous disease with variable clinical features and patients can be stratified in different risk groups, according to their clinical and biochemical parameters.

The international prognostic index (IPI) remains the most used tool to predict response to treatment [2], but does not reflect DLBCL molecular heterogeneity within each prognostic subgroup. Gene expression profiling (GEP) studies showed that DLBCL can be further classified in distinct molecular categories on the basis of the cell of origin (COO) [3]: germinal center B-cell (GCB) subtype, activated B-cell (ABC) subtype, and primary mediastinal B-cell (PMBL) subtype. The GCB subtype is significantly associated with a better overall survival (OS), whereas the ABC subgroup has a poorer outcome. The Hans algorithm is widely used in the routine practice to segregate DLBCL in two subgroups, germinal center (GC) and non-germinal center (non-GC), that match the GCB and ABC molecular entities, respectively [4, 5]. Other studies based on GEP and cytogenetic approaches have investigated DLBCL biology with the aim of improving patients risk stratification [6–11]. Moreover, as about one third of patients have either refractory disease or relapse after the initial therapy, chemoresistance is a challenge for DLBCL management. Therefore, new prognostic markers and new therapeutic approaches to improve the long-term outcome are needed.

Alteration of the epigenetic regulation (e.g., DNA methylation and histone modifications) of gene expression is a hallmark of cancer [12]. Changes in the expression of epigenetic regulators, such as enhancer of zeste 2 polycomb repressive complex 2 subunit (EZH2), myeloid/lymphoid or mixed-lineage leukemia (MLL), isocitrate dehydrogenase 2 (NADP+) mitochondrial (IDH2), tet methylcytosine dioxygenase 2 (TET2) and DNA (cytosine-5-)-methyltransferase 3 alpha (DNMT3A), have been described in hematologic malignancies, including DLBCL, and can have a prognostic value [12–15]. Of note, epigenetic alterations can be reversed by pharmacological drugs, such as histone deacetylase (HDAC), DNA methyltransferase (DNMT), or histone methyltransferase (HMT) inhibitors, and they are currently used in the clinic or tested in clinical trials in patients with relapsed or refractory DLBCL [16–19].

The aim of this study was to identify prognostic factors that allow the stratification of patients with DLBCL in different risk groups, based on the gene expression profile of epigenetic regulators. We report the design of a new risk score (EpiScore) that classifies patients with DLBCL in high, intermediate and low risk and highlight pathways that could be involved in DLBCL poor prognosis.

## RESULTS

### Prognostic value of epigenetic genes in DLBCL

**Figure 1:**
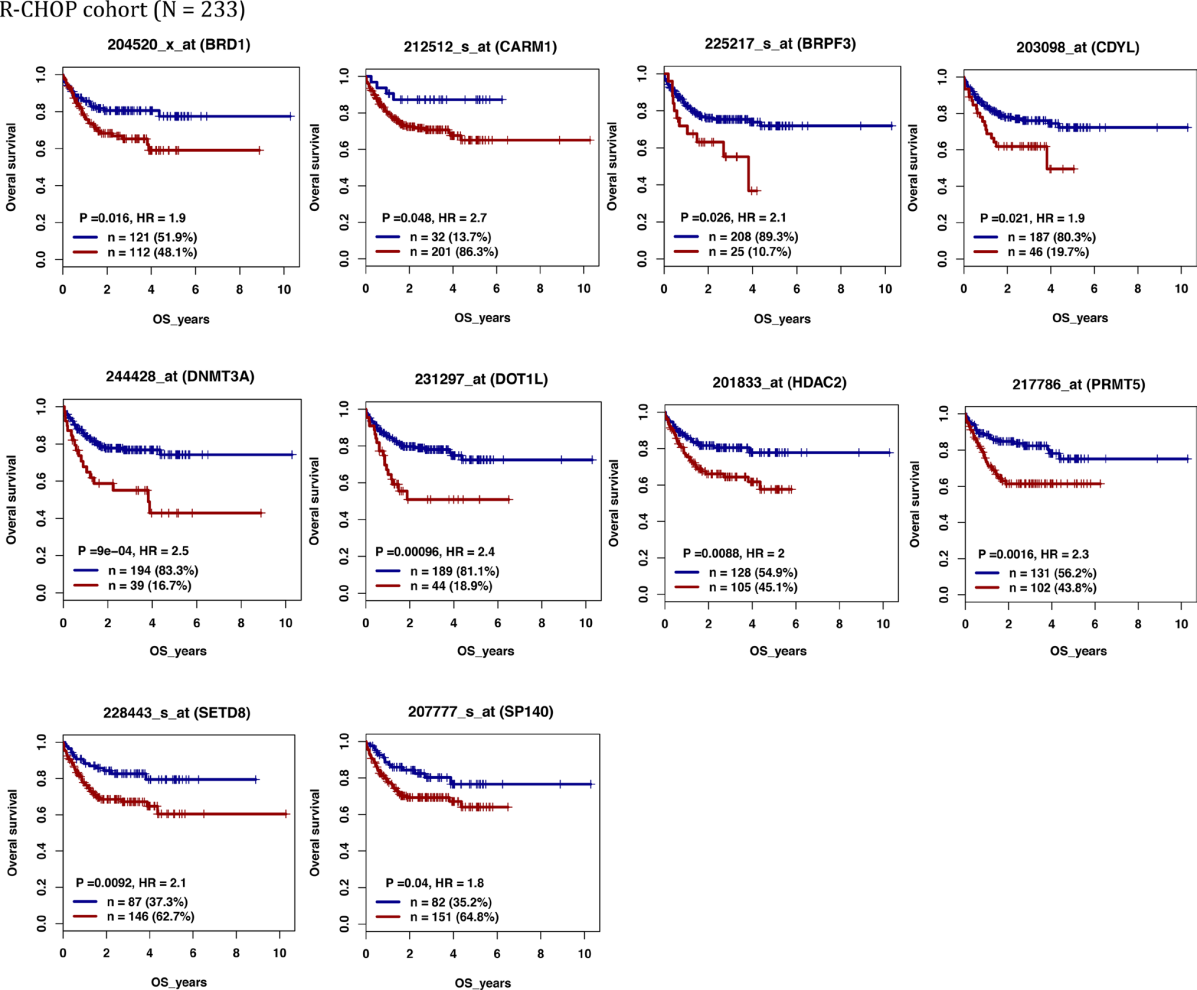
Epigenetic regulators with prognostic value in patients with DLBCL (Lenz R-CHOP cohort *n* = 233) For a given gene, a prognostic expression cut-off was calculated using the Maxstat algorithm, as described in Materials and Methods, to split patients in two groups (high and low risk) according to their overall survival (OS). *BRD1*: bromodomain containing 1; *CARM1*: coactivator associated arginine methyltransferase 1; *BRPF3*: bromodomain and PHD finger containing 3; *CDYL:* chromodomain Y like; *DNMT3A:* DNA cytosine-5-methyltransferase 3 alpha; *DOT1L*: DOT1-like histone H3K79 methyltransferase; HDAC2: histone deacetylase 2; *PRMT5:* protein arginine methyltransferase 5; *SETD8*: also known as KMT5 lysine (K)-specific methyltransferase 5A; *SP140*: SP140 nuclear body protein.

**Figure 2:**
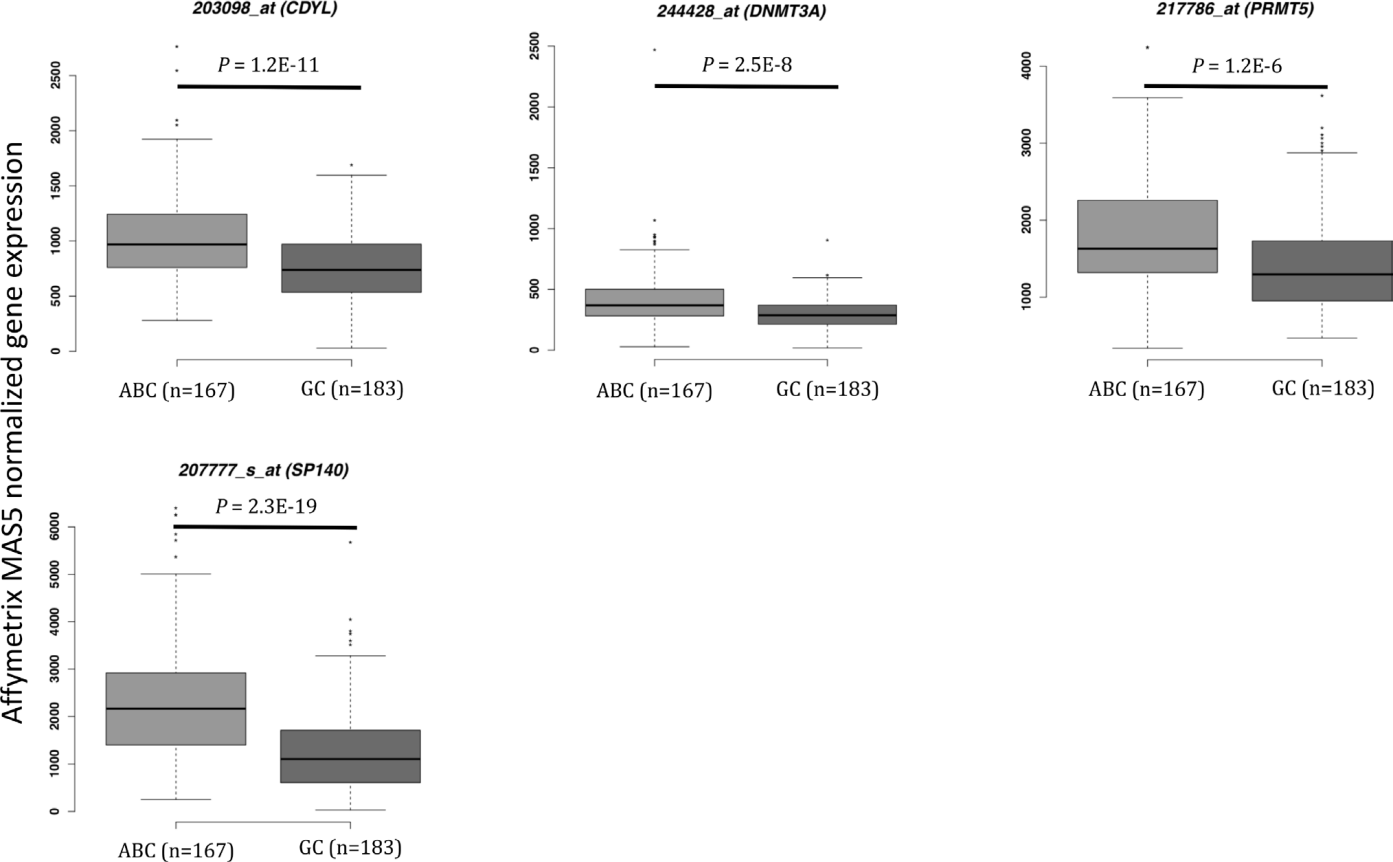
Epigenetic gene expression in the ABC and GC DLBCL subgroups (Lenz R-CHOP and CHOP cohorts) *CDYL:* chromodomain Y-like; *DNMT3A:* DNA cytosine-5-methyltransferase 3 alpha; *DOT1L*: DOT1-like histone H3K79 methyltransferase; *PRMT5:* protein arginine methyltransferase 5; *SP140*: SP140 nuclear body protein; ABC: activating B cell; GC: germinal center. Results were compared using Student *t* test. The box-plot diagrams included the median value and the interquartile rage (IQR). The error bars represent the minimum for the values under the median and the outliers are identified as the third quartile plus 1.5 IQR (SPSS software).

We defined as epigenetic genes, genes belonging to the following families: DNMTs, methyl-CpG-binding domain (MBD) proteins, histone acetyltransferases (HATs), HDACs, HMTs, histone demethylases and bromodomain (BRD) and extra-terminal motif (BET) proteins [20] (Supplementary Table 1). We then investigated the prognostic value of their expression in DLBCL in two independent cohorts of patients with newly-diagnosed DLBCL (accession number GSE10846 [21]): 233 patients treated with R-CHOP (rituximab-cyclophosphamide, hydroxydaunorubicin, vincristine, and prednisone) (Lenz R-CHOP cohort) used as training cohort and 181 patients treated with CHOP (cyclophosphamide, hydroxydaunorubicin, vincristine, and prednisone) (Lenz CHOP cohort) used as validation cohort. Using the Maxstat R function and Benjamini-Hochberg multiple testing correction [22, 23], we found that ten probe sets had a prognostic value for OS (adjusted *P* value < 0.05) in both cohorts (Figure 1, Supplementary Figure 1 and Table 1A). Analysis of the expression of these ten prognostic genes in the ABC and GCB molecular subgroups showed that four were significantly overexpressed in the ABC subgroup: SP140 nuclear body protein (*SP140*) (2.3.10^–19^), chromodomain Y like (*CDYL*) (1.2.10^–11^), *DNMT3A* (2.5.10^–8^) and protein arginine methyltransferase 5 (*PRMT5*) (1.2.10^–6^) (Figure 2).

**Table 1:** Cox univariate and multivariate analyses of overall survival in patients with diffuse large B-cell lymphoma (Lenz R-CHOP cohort, *n* = 233)

A.	Overall survival (*n* = 233)	
Epigenetic regulator	HR	*p* value
BRD1	1.9	0.01
BRPF3	2.1	0.02
CARM1	2.7	0.04
CDYL	1.9	0.02
DNMT3A	2.5	<0.0001
DOT1L	2.4	0.0009
HDAC2	2	0.008
PRMT5	2.3	0.001
SETD8	2.1	0.009
SP140	1.8	0.04

B.	Overall survival (*n* = 233)	
Epigenetic regulator	HR	*p* value
BRD1	1.4	NS
BRPF3	1.2	NS
CARM1	1.15	NS
CDYL	1.56	NS
DNMT3A	2.62	0.001
DOT1L	2.03	0.01
HDAC2	1.67	NS
PRMT5	1.58	NS
SETD8	2.0	0.03
SP140	1.45	NS

The indicated prognostic factors were tested as single variables (A) or multi-variables (B) using a Cox-model. *P*-values and hazard ratios (HR) are shown. NS: not significant at the 5% threshold.

Finally, using multivariate Cox analysis, we found that three of these ten epigenetic genes remained independent prognostic factors: DOT1-like histone H3K79 methyltransferase (*DOT1L*), *SETD8* (also known as lysine (K)-specific methyltransferase 5A, *KMT5*) and *DNMT3A* (Table 1B).

### DNMT3A and DOT1L protein expression in patients with DLBCL

**Figure 3:**
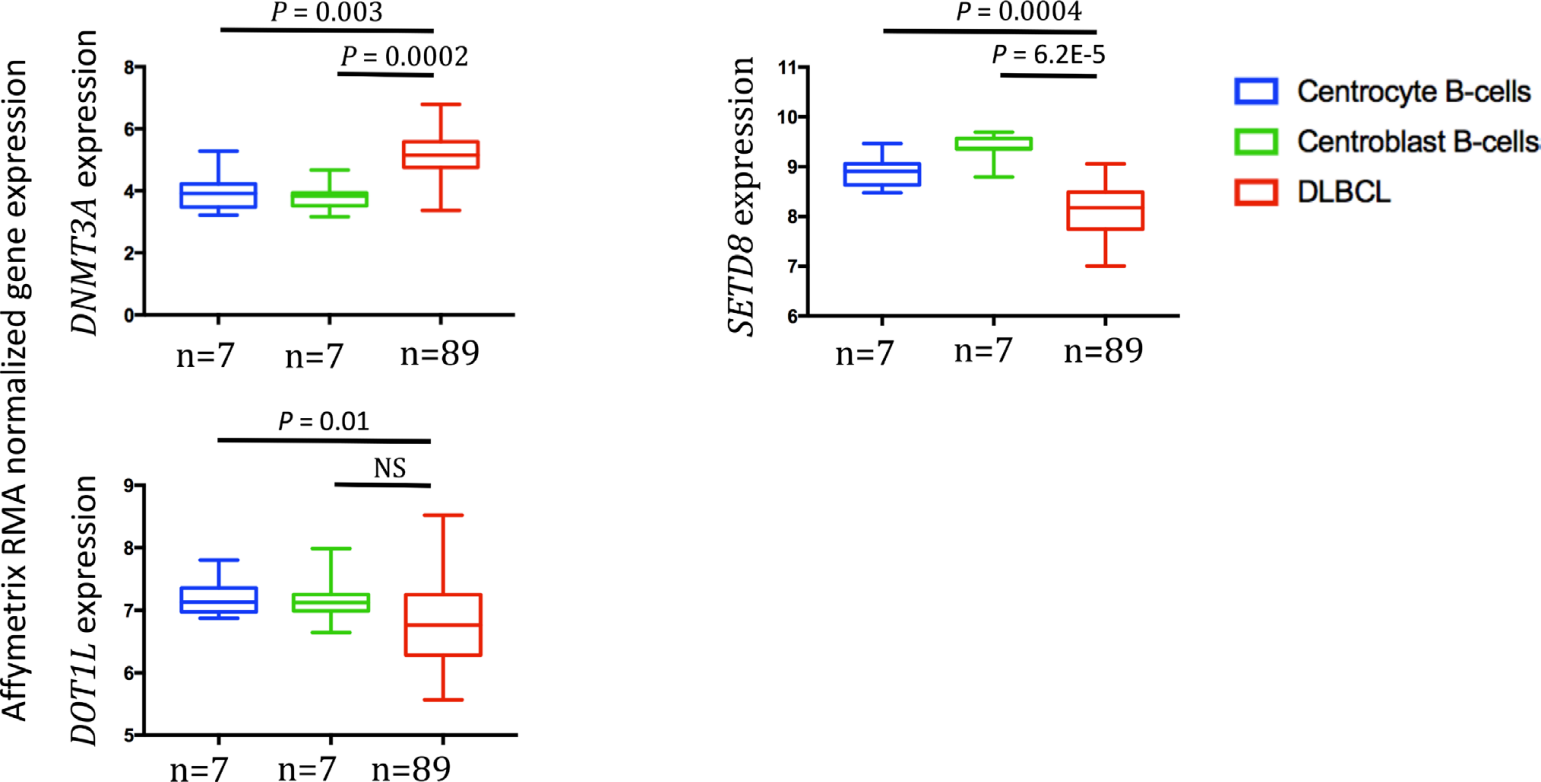
*DNMT3A*, *SETD8* and *DOT1L* gene expression in DLBCL samples compared with normal centrocytes and centroblasts (GSE56315 dataset) Results were compared using Student *t* test. The box-plot diagrams included the median value and the interquartile rage (IQR). The error bars represent the minimum for the values under the median and the outliers are identified as the third quartile plus 1.5 IQR (SPSS software).

We then compared *DNMT3A*, *SET8* and *DOT1L* gene expression in normal centrocytes (*n* = 7), normal centroblasts (*n* = 7) and DLBCL samples (*n* = 89) [24]. DNMT3A was significantly overexpressed in DLBCL samples compared with normal centrocytes (*p* = 0.003) and centroblasts (*p* = 0.0002) (Figure 3). Conversely, SETD8 and DOT1L were downregulated in DLBCL compared with normal centrocytes (*p* = 0.0004 and *p* = 0.01 respectively) and centroblasts (*p* = 6.2.10^–5^ and not significant, respectively) (Figure 3). *DOT1L* and *SETD8* gene expression was also validated by RT-qPCR using 10 DLBCL cell lines (Supplementary Figure 4).

**Figure 4:**
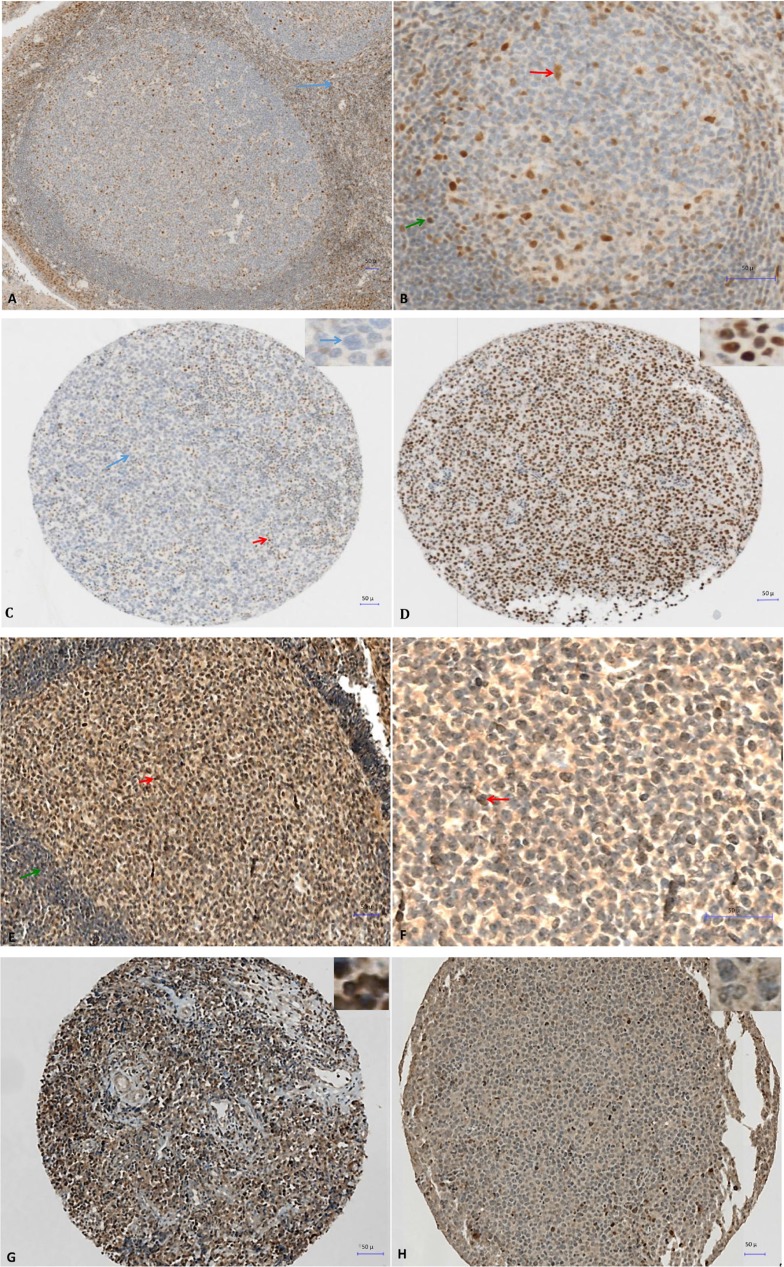
Immunohistochemical analysis of DNMT3A expression in tonsillar lymphoid tissue (**A** and **B**) and in two patients with DLBCL at diagnosis (**C** and **D**) and immunohistochemical analysis of DOT1L expression in tonsillar lymphoid tissue (**E** and **F**) and in two patients with DLBCL at diagnosis (**G** and **H**). (A) Nuclear expression of DNMT3A in reactive T cells of the interfollicular region (blue arrow) in a reactive human tonsil sample. (B) Nuclear expression of DNMT3A in some naive B cells in the mantle zone (green arrow), and in tingible-body macrophages and follicular dendritic cells in the germinal center (red arrow) in a reactive human tonsil sample. (C) Immunohistochemical analysis of DNMT3A expression in a patient with DLBCL (IPI 3). The patient was in complete remission after treatment with R-CHOP (six cycles) without relapse after 2 years of follow-up. No expression in large tumoral B cells (blue arrow and inset) and nuclear expression in reactive T cells show (red arrow). (D) Strong nuclear expression of DNMT3A in malignant B cells (see also inset) in a patient with DLBCL (IPI 3) with disease progression despite R-CHOP treatment. The patient died of the disease 6 months the after initial diagnosis. (E–F) Nuclear expression of DOT1L in some centrocytes and centroblasts in the germinal center (red arrow) in a reactive human tonsil sample whereas naive B cells in the mantle zone (green arrow) are negative. (G) Strong nuclear expression of DOT1L in a patient with DLBCL (80% of tumor cells are positive). (H) Low nuclear expression of DOT1L in another patient with DLBCL (only 2% of tumor cells are positive).

To assess DNMT3A, SETD8 and DOT1L protein expression we selected four DLBCL cell lines with different *DNMT3A*, *SETD8* and *DOT1L* gene expression: DB (high *DNMT3A* and *SETD8* expression, low *DOT1L* expression), NUDUL1 (high *DNMT3A* and *SETD8* expression), RI1 (high *DNMT3A* and *DOT1L* expression, low *SETD8* expression) and SUDHL5 (low *DNMT3A* expression). Incubation of formalin-fixed, paraffin-embedded cell pellets with anti-DNMT3A, -SETD8 or -DOT1L antibodies showed that anti-SETD8 antibody gave only non-specific staining in positive and negative controls (data not shown). Conversely, we detected DNMT3A nuclear expression in RI1 (35%) and NUDUL1 (10%), DB (5%) cells, (strong *DNMT3A* gene expression), but not in SUDHL5 cells (low *DNMT3A* expression) (Supplementary Figure 2). DOT1L nuclear expression was detected in RI1 (80%), SUDHL5 (40%) and NUDUL1 (20%) but not in DB cell line, confirming gene expression data (Supplementary Figure 2). Based on these data, we then investigated the prognostic value of DNMT3A and DOT1L protein expression in samples from 31 patients with DLBCL treated with R-CHOP or R-CHOP-like therapy and in five non-neoplastic tissues (two reactive lymph nodes and three tonsil specimens) as control (all from the Pathology Department, Montpellier University Hospital, France). In agreement with the microarray data, DNMT3A and DOT1L showed variable expression patterns. In tonsils and reactive lymph nodes, DNMT3A was expressed in the nucleus of some naive B cells in the mantle zone while GC B cells were negative (Figure 4A and 4B) whereas DOT1L was expressed in some centrocytes and centroblates in the GC while naïve B cells in the mantle zone did not show any expression (Figure 4E and 4F). In DLBCL samples, the percentage of DNMT3A-positive tumor cells varied between 0% and 100% (Figure 4C and 4D) and the percentage of DOT1L-positive tumor cells between 1% and 85% (Figure 4G and H) (Supplementary Figure 5).

**Figure 5:**
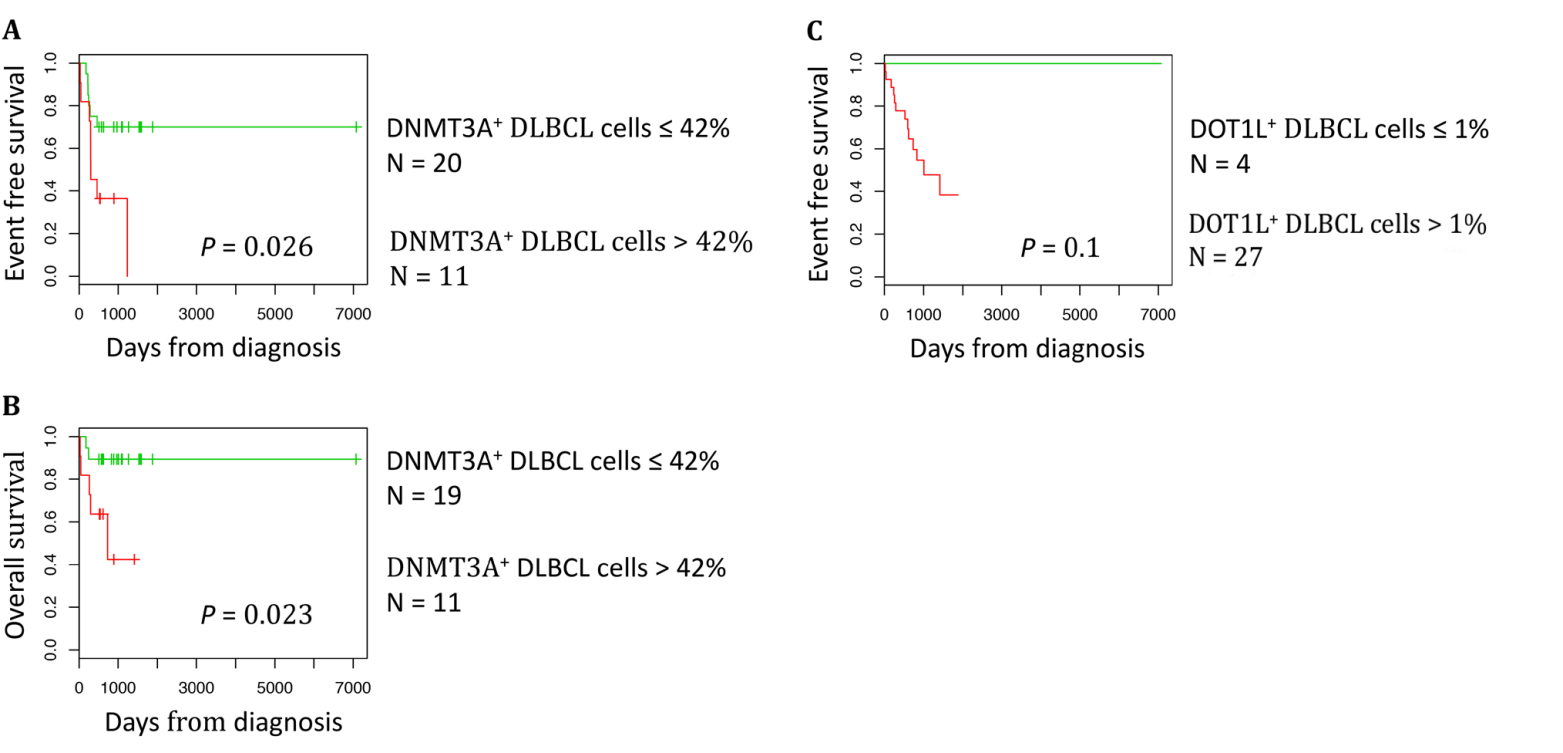
DNMT3A protein expression is a prognostic factor in DLBCL The prognostic information provided by DNMT3A protein expression was investigated using the Maxstat algorithm. A cut-off of 42% of stained tumor cells allowed splitting the patients in a high risk and low risk group (**A**) Event free survival, (**B**) Overall survival. The prognostic information provided by DOT1L protein expression was investigated using the Maxstat algorithm. A cut-off of 1% of stained tumor cells allowed splitting the patients in EFS high risk and low risk groups (**C**).

To determine whether DNMT3A and DOT1L protein expression are associated with a prognostic value, we ranked the 31 DLBCL samples according to their DNMT3A or DOT1L protein expression. Using the Maxstat R function [22, 23], that allow to determine the optimal cutpoint for continuous variables, we found that the maximum difference in OS and event-free survival (EFS) was obtained using a cut-off of 42% of DNMT3A-positive tumor cells that split patients in two groups (high and low risk) (Figure 5A and 5B). Concerning DOT1L protein expression, we identified a trend (*P* = 0.1) for an association with no detectable DOT1L protein expression and a better EFS in the cohort of 31 DLBCL patients tested (Figure 5C). Validation of the prognostic value of DOT1L protein expression in a larger cohort of patients will be of interest.

We then studied the correlation between DNMT3A protein overexpression (defined as ≥42% of DNMT3A-positive tumor cells) or DOT1L protein expression (defined as > 1% of DOT1L-positive tumor cells) and the usual clinicopathological parameters (gender, age, Ann-Arbor clinical stage, lactate dehydrogenase serum level, performance status and the IPI score) in the 31 patients with DLBCL. We did not find any significant correlation between DNMT3A overexpression or DOT1L expression and any of the clinical parameters analyzed (Table 2). On the other hand, when we took into account the tumor expression profile, DNMT3A overexpression was significantly correlated with BCL2 protein expression (*P* = 0.0261). Conversely, DNMT3A expression status was not correlated with the GC or non-GC profile, or with P53, MYC and KI67 expression (Table 2A). No significant correlation between DOT1L expression and the phenotypic parameters was identified (Table 2B).

**Table 2A:** Association between DNMT3A protein overexpression and clinicopathological variables in patients with diffuse large B-cell lymphoma (*n* = 31)

Clinicopathlogical variables		Total	DNMT3A overexpression
		n^*^	*n* (%)^**^
Sex			
Men		15	6 (40)
Women		16	6 (35.5)
	*p*-value		1.000
Age (years)			
≤60		14	6 (42.9)
>60		17	6 (35.3)
	*p*-value		0.7241
Stage (Ann Arbor)			
I/II		10	2 (20)
III/IV		16	9 (56.25)
	*p*-value		0.1092
Serum LDH			
Normal		7	3 (42.9)
Elevated		10	4 (40)
	*p*-value		
Performance status			
<2		21	9 (42.9)
≥2		2	0 (0)
	*p*-value		0.5020
IPI score^***^			
Low		23	8 (34.8)
High		8	4 (50)
	*p*-value		0.6757
Response to first-line treatment			
CR		23	7 (30.4)
PR/PD		7	4 (57.1)
	*p*-value		0.3717
P53 expression			
Yes		9	2 (22.2)
Not		22	10 (45.5)
	*p*-value		0.4184
BCL2 expression			
Yes		24	12 (50)
Not		7	0 (0)
	*p*-value		0.0261^**^
MYC expression			
Yes		6	3 (50)
Not		25	9 (36)
	*p*-value		0.6526
KI67 expression			
≤80		23	10 (43.5)
>80		8	2 (25)
	*p*-value		0.4325
GCB immunophenotype			
GCB phenotype		16	7 (43.75)
Non-GCB phenotype		15	5 (33.3)
	*p*-value		0.7160

DNMT3A protein overexpression was defined as ≥ 42% of DNMT3A-positive tumor cells in the sample. ^*^The total number in some categories was lower than that of the whole sample because of missing clinical data. ^**^The number in bold indicates significant correlation (*p* < 0.05) (name of statistical test). ^***^Low scores include low and low-intermediate IPI scores; high scores include intermediate-high and high IPI scores. The IPI groups were defined as follows: low risk group = IPI score 0 or 1, low-intermediate risk group = IPI score 2, high-intermediate risk group = IPI score 3, and high risk group = IPI score 4 or 5. LDH: lactate dehydrogenase; IPI: International Prognostic Index; CR: complete response; PR: partial response; PD: progressive disease; GCB: germinal center B-cell like.

**Table 2B:** Association between DOT1L protein overexpression and clinicopathological variables in patients with diffuse large B-cell lymphoma (*n* = 31)

Clinicopathological variables		Total	DOT1L positive expression
		n^*^	*n* (%)^**^
Sex			
Men		15	14 (93.3)
Women		16	14 (87.5)
	*p*-value		1.000
Age (years)			
≤60		14	13 (92.9)
>60		17	15 (88.2)
	*p*-value		1.000
Stage (Ann Arbor)			
I/II		10	8 (80)
III/IV		16	15 (93.75)
	*p*-value		0.5385
Serum LDH			
Normal		7	6 (85.7)
Elevated		10	8 (80)
	*p*-value		1.000
Performance status			
<2		21	19 (90.5)
≥2		2	2(100)
	*p*-value		1.000
IPI score^***^			
Low		23	21 (91.3)
High		8	7 (87.5)
	*p*-value		1.000
Response to first-line treatment			
CR		23	20 (87)
PR/PD		7	7 (100)
	*p*-value		1.000
P53 expression			
Yes		9	9 (100)
Not		22	19 (86.4)
	*p*-value		0.5375
BCL2 expression			
Yes		24	22 (91.7)
Not		7	6 (85.7)
	*p*-value		0.5497
MYC expression			
Yes		6	6 (100)
Not		25	22 (88)
	*p*-value		1.000
DNMT3A overexpression			
Yes		12	12 (100)
Not		19	16 (84.2)
	*p*-value		0.2645
KI67 expression			
≤80		23	21 (91.3)
>80		8	7 (87.5)
	*p*-value		1.000
GCB immunophenotype			
GCB phenotype		16	14 (87.5)
Non-GCB phenotype		15	14 (93.3)
	*p*-value		1.000

DOT1L protein expression was defined as > 1% of DOT1L-positive tumor cells in the sample. DNMT3A protein overexpression was defined as ≥ 42% of DNMT3A-positive tumor cells in the sample.^*^The total number in some categories was lower than that of the whole sample because of missing clinical data. ^**^The number in bold indicates significant correlation (*p* < 0.05) (name of statistical test). ^***^Low scores include low and low-intermediate IPI scores; high scores include intermediate-high and high IPI scores. The IPI groups were defined as follows: low risk group = IPI score 0 or 1, low-intermediate risk group = IPI score 2, high-intermediate risk group = IPI score 3, and high risk group = IPI score 4 or 5. LDH: lactate dehydrogenase; IPI: International Prognostic Index; CR: complete response; PR: partial response; PD: progressive disease; GCB: germinal center B-cell like.

### EpiScore prognostic value in DLBCL

**Figure 6:**
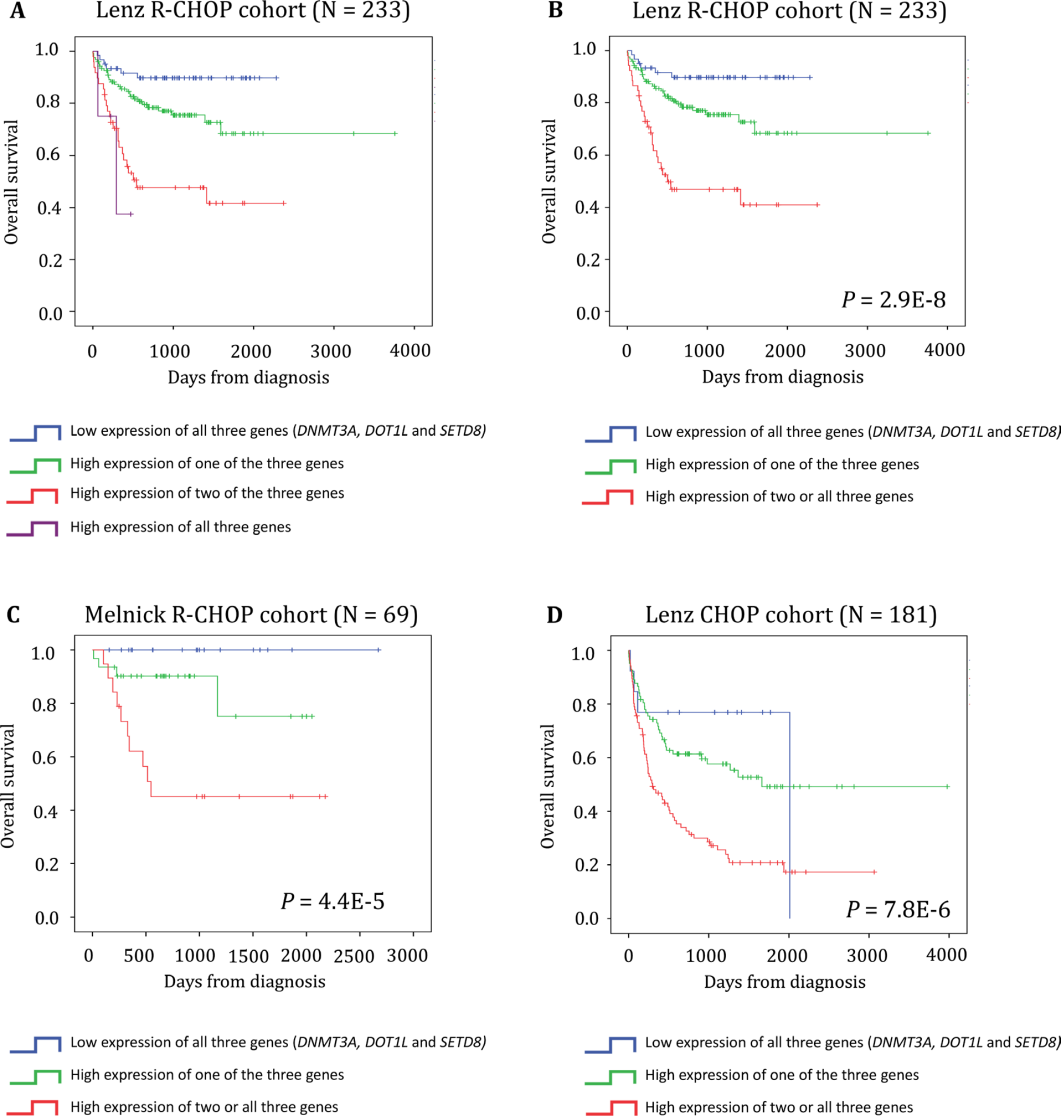
EpiScore predicts the overall survival in patients with DLBCL (**A**) Kaplan-Meier estimates of the overall survival in patients from the Lenz R-CHOP cohort subdivided in four groups on the basis of *DNMT3A, DOT1L* and *SETD8* gene expression. When two consecutive groups showed no significant difference, they were merged. (**B**) This process led to the identification of three groups: low risk (low expression of *DNMT3A, DOT1L* and *SETD8*; blue), intermediate risk (high expression of one of these three genes; green) and high risk (high expression of two or all three genes; red). (**C–D**) EpiScore prognostic value at diagnosis was confirmed in two other independent cohorts.

We then used the three genes (*DNMT3A, SET8* and *DOT1L*) identified as independent prognostic factors in patients with DLBCL to develop a risk score (EpiScore) based on their expression level. To this aim, we split the training cohort (Lenz R-CHOP cohort, *n* = 233 patients) in four groups according to the tumor expression of these three genes: group 1 (low *DNMT3A, DOT1L* and *SETD8* expression), group 2 (high expression of one of the three genes), group 3 (high expression of two of the three genes), and group 4 (high expression of all three genes). When the Kaplan Meier analysis did not show any significant OS difference between consecutive groups, we merged the two groups (Figure 6A). According to that, the group 3 (high expression of two of the three genes) and the group 4 (high expression of all three genes) were merged. This approach resulted in three groups with different OS values. Group 1 (27% of patients; low risk, low *DNMT3A*, *DOT1L* and *SETD8* expression) and group 2 (51.9% of patients; intermediate risk, high expression of one of the three genes) did not reach the median OS; conversely, group 3 (22.4% of patients; high risk, high expression of two or all three genes) had a median OS of 16.5 months (Figure 6B). We then validated the EpiScore prognostic value in two independent cohorts of patients with DLBCL (Melnick cohort: *n* = 69 patients treated with R-CHOP, *P* = 4.4E-5, *n* = 69; and Lenz CHOP cohort: *n* = 181 patients, *P* = 7.8E-6) (Figure 6C and 6D).

We then investigated whether EpiScore provided additional prognostic information compared with previously identified, poor outcome-related factors, such as the GCB and ABC molecular subgroups, age, IPI, Gene Expression-based Risk Score (GERS) [11] and DNA repair score [25]. First, we confirmed using Cox univariate analysis that EpiScore, GERS, age, GCB-ABC molecular subgroups, IPI and DNA repair score had a prognostic value in the Lenz R-CHOP cohort (*n* = 233 patients) (*P* < 0.0001, Table 3A). In two by two comparisons, EpiScore tested with GERS, age, GCB-ABC molecular subgroups, IPI or DNA repair score remained significant (*P* < 0.0001, *P* = 0.001, *P* = 0.001, *P* < 0.0001 and *P* < 0.0001 respectively, Table 3B). Conversely, when we tested all parameters together, only EpiScore, GERS and DNA repair score retained their prognostic values (Table 3C).

**Table 3:** Cox univariate and multivariate analyses of overall survival in patients with diffuse large B-cell lymphoma (Lenz R-CHOP cohort, *n* = 233)

A.	Overall survival (*n* = 233)	
Prognostic variable	**HR**	***p* value**
GERS	5.49	<0.0001
Age (>60 years)	2.2	<0.0001
GCB-ABC molecular subgroups	2.75	<0.0001
IPI	1.79	<0.0001
DNA repair score	3.87	<0.0001
EpiScore	3.64	<0.0001

B.	Overall survival (*n* = 233)	
EpiScore	2.49	<0.0001
GERS	4.73	<0.0001
EpiScore	2.82	<0.0001
Age (>60 years)	2.61	0.001
EpiScore	2.29	<0.0001
GCB-ABC molecular subgroups	2.80	0.001
EpiScore	3.20	<0.0001
IPI	1.61	<0.0001
EpiScore	1.67	0.02
DNA repair score	2.47	<0.0001

C.	Overall survival (*n* = 233)	
Prognostic variable	HR	*p* value
GERS	3.77	.001
Age (>60 years)	1.62	NS
GCB-ABC molecular subgroups	1.67	NS
IPI	1.29	NS
DNA repair score	3.18	<0.0001
EpiScore	1.81	0.04

The indicated prognostic factors were tested as single variables (A), multi-variables two by two (B) or multivariate with all variables (C) using a Cox-model. *P*-values and hazard ratios (HR) are shown. NS: not significant at the 5% threshold. The IPI groups were defined as follows: low risk group = IPI score 0 or 1, low-intermediate risk group = IPI score 2, high-intermediate risk group = IPI score 3, and high-risk group = IPI score 4 or 5.

We previously reported deregulated DNA repair pathways in DLBCL to develop novel strategies exploiting the concept of synthetic lethality and overcome drug resistance [26]. We investigated the type of DNA repair pathways deregulated in the different subgroups delineated by the EpiScore. Interestingly, we identified a significantly higher value of the Non-Homologous-End-Joining (NHEJ), FANC, Nucleotide Excision Repair (NER), Base Excision Repair (BER), Homologous Recombination Repair (HRR) and Mismatch Repair (MMR) scores in the EpiScore defined high-risk compared to low-risk patients (Supplementary Figure 6).

### Tumor cells from patients in the EpiScore high-risk group (group 3) have a HDAC gene signature

Finally, we compared the gene expression profiles of tumors from patients of the Lenz R-CHOP cohort who were included in the EpiScore high risk group (*n* = 52) or low risk group (*n* = 60) by gene set enrichment analysis (GSEA). Genes related to the Class I HDAC pathway (PID_HDAC_CLASSI_PATHWAY, *P* = 0.0001 and KASLER_HDAC7_TARGETS_1_UP, *P* = 0.002), proliferation (LIN_APC_TARGETS, *P* < 0.0001) and MTOR pathway (PID_MTOR_4PATHWAY, *P* < 0.0001) were significantly enriched in the EpiScore high risk group compared with the low risk group (Supplementary Figure 4 and Supplementary Tables 2 to 5).

**Figure 7:**
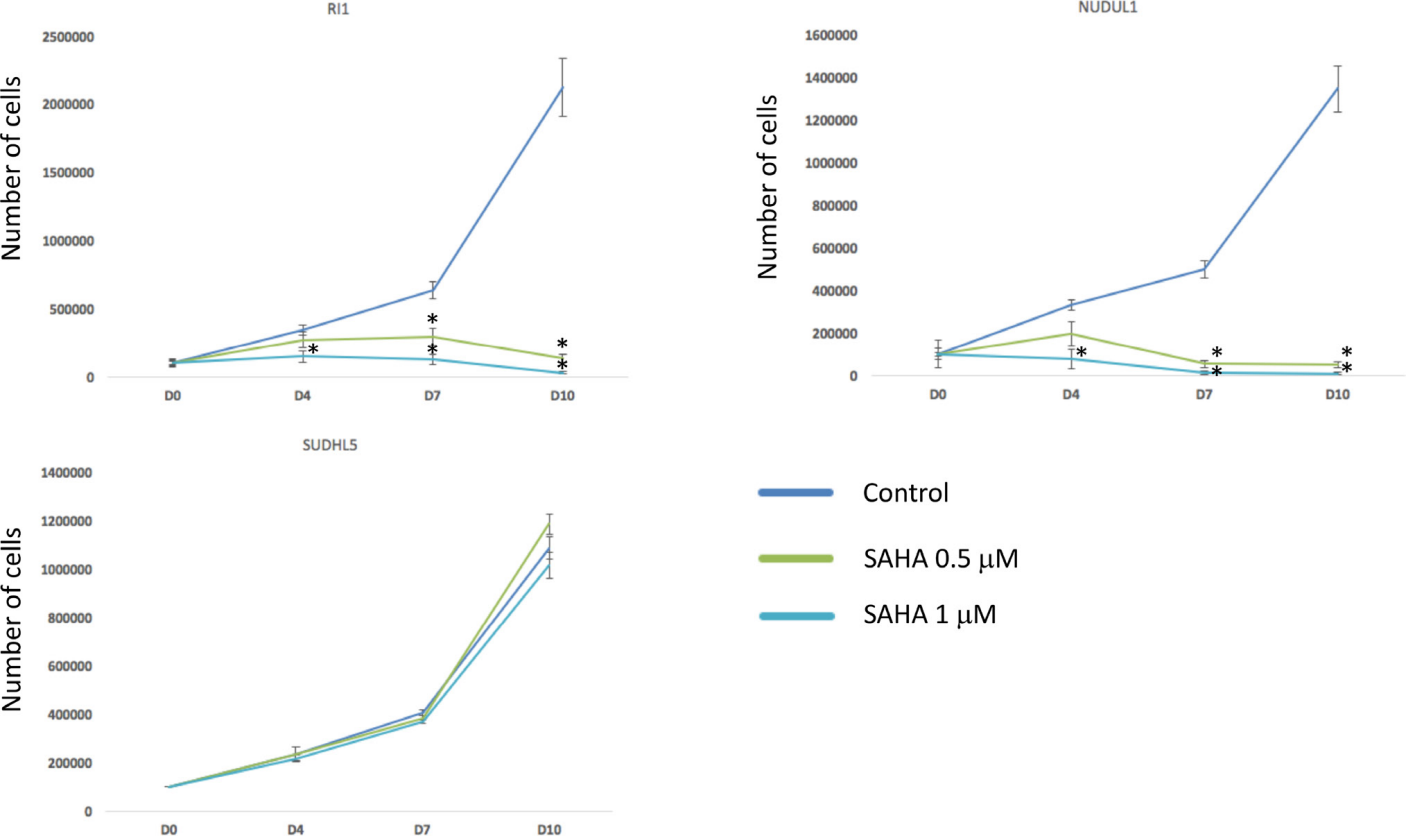
HDACi treatment induces toxicity in cell lines overexpressing EpiScore genes DLBCL cell lines overexpressing 2 out of the 3 genes of the EpiScore (RI1 and NUDUL1) and SUDHL5, characterized by low expression of the 3 genes, were cultured for 10 days without drug (control), with 0.5 µM or with 1 µM of SAHA and cell viability was analyzed by trypan blue assay. Data are representative of three idependent experiments. Statistical significance was tested using a Wilcoxon test for pairs (^*^P < 0.05).

According to these data, we compared the response to HDACi (SAHA) of two DLBCL cell lines overexpressing 2 out of the 3 genes (RI1 and NUDUL1) with SUDHL5, characterized by low expression of the 3 genes. Interestingly, SAHA induced a significant inhibition of RI1 and NUDUL1 cell growth (*P* < 0.05) (Figure 7). SAHA treatment has no significant effect on SUDHL5 cell growth (Figure 7).

Altogether, these data suggest that DLBCL patients with high-risk EpiScore are associated with a HDAC GEP signature and could benefit from HDACi targeted treatment.

## DISCUSSION

In the present study, we assessed the prognostic value of epigenetic genes in DLBCL and built a risk score (EpiScore) based on the expression of three of them. EpiScore allowed splitting patients with DLBCL in three risk groups: group 1 (low risk, low expression of *DNMT3A*, *DOT1L* and *SETD8*), group 2 (medium risk, high expression of one of these three genes), and group 3 (high risk, high expression of two or all three genes). We then show that EpiScore is an independent predictor factor for OS when compared with the previously published prognostic factors.

Besides the powerful prognostic value of EpiScore, the present study highlights pathways that could be involved in poor DLBCL prognosis. Among the epigenetic regulators with a prognostic value for OS, *DNMT3A* encodes a DNA metyltransferase that catalyzes de novo DNA methylation. DNA methylation is not only involved in lymphomagenesis (DNA methylation is altered in lymphomas compared with normal B cells) [27], but also in lymphoma progression and relapse. Moreover, *Pan et al.* showed that in DLBCL, intra-tumor methylation heterogeneity at diagnosis is predictive of relapse occurrence and that intra-tumour methylation heterogeneity decreases at relapse, consistent with clonal selection upon chemotherapy treatment [28]. They also reported a methylation signature, based on differentially methylated regulatory elements between DLBCL at diagnosis and at relapse. This signature comprises genes involved in lymphoma progression, including the TGF-β receptor pathway known to be associated with relapse and chemoresistance in DLBCL [15, 29]. Thus, aberrant DNA methylation in DLBCL might contribute to chemoresponsiveness. Mutations in *DNMT3A* have been reported in various hematologic malignancies, including acute myeloid leukemia (AML), myelodysplastic syndrome (MDS) and T-cell lymphoma and leukemia [30–34], underlining a potent tumor suppressor role. Furthermore some studies have demonstrated that *DNMT3A* mutations may represent an early event in the development of these malignancies [35, 36]. In normal hematopoiesis, *DNMT3A* silences self-renewal genes in hematopoietic stem cells (HSCs) and facilitates hematopoietic differentiation [30]. Moreover, *DNMT3A* mutations have an adverse prognostic impact in hematologic cancers [37, 38]. In agreement, we found that high DNMT3A protein expression is associated with poorer OS and EFS in 31 patients with DLBCL. We also identified, for the first time, a significant correlation between BCL2 and DNMT3A protein expression in DLBCL samples. Analyses of the biological links between BCL2 and DNMT3A expression in DLBCL will be of interest to define if DNMT3A could play a role in BCL2 deregulation in DLBCL. Two other studies have shown that DNMTs (DNMT1, DNMT3A and DNMT3B) are involved in DLBCL pathogenesis of, but only DNMT1 and DNMT3B appeared to be adverse prognostic factors [39, 40]. Taken together, these data suggest that *DNMT3A* could be involved in DLBCL lymphomagenesis. Furthermore, epigenetic therapy using 5-azacytidine (Aza) and 5-aza-2’deoxycytidine (decitabine) has proved to be a successful treatment strategy in hematologic cancers, especially MDS and AML [41]. *DNMT3A* mutations in patients with MDS is also an independent prognostic factor of a better response to Aza treatment [42]. Another study reported that in AML, Aza and decitabine can reverse methylation and silencing of a series of genes and that their reactivation may contribute to the therapeutic activity of both drugs [43]. Decitabine also exhibits strong antineoplastic activity in anaplastic large cell lymphoma (ALCL), inducing apoptosis, cell death and cell cycle arrest both *in vitro* and *in vivo* [44]. Of interest, in DLBCL cell lines, DNMT inhibitors (DNMTi) enhance the response to conventional chemotherapy and can reprogram chemoresistant cells to regain chemosensitivity [15]. Furthermore, a phase 1 clinical trial that evaluated DNMTi in combination with standard immunochemotherapy in newly diagnosed patients with high-risk DLBCL reported a high rate of complete remission, highlighting DNMTi chemosensitization effect [15]. Altogether these data suggest that DNMTi could constitute an interesting therapeutic approach for patients with DLBCL included in the EpiScore high risk group.

*DOT1L* and *SETD8* are the other two genes included in the EpiScore. *DOT1L* encodes a HMT that methylates lysine-79 of histone H3, involved in the regulation of various cellular processes, such as development, reprogramming, differentiation or proliferation, and controls the development of diseases, including leukemia [45–48]. DOT1L inhibition is beneficial in MLL-fusion-induced leukemia and DOT1L inhibitors (DOT1Li) are under investigation in a phase 1 clinical trial in patients with this pathology [48–50]. Interestingly, DOT1L is also involved in DNA damage response and repair and its inhibition can reverse chemoresistance of MLL-rearranged leukemic cells [45, 46, 51]. This suggests that DOT1Li might impair the DNA damage and repair pathway and thus, sensitize MLL-rearranged leukemic cells to chemotherapy. As chemoresistance is challenging in the treatment of DLBCL, DOT1Li could represent an interesting therapeutic strategy in high-risk DLBCL characterized by high DOT1L expression. *SETD8* encodes the sole lysine methyltransferase that catalyzes monomethylation of histone H4 lysine 20 (H4K20me1). *SETD8* is involved in various important biological processes, including DNA replication, cellular proliferation and development, chromosome condensation and activation of DNA replication checkpoints [52, 53]. Particularly SETD8 promotes DNA double strand break (DSB) repair and loss of SETD8 results in massive DNA damage, cell cycle arrest and induction of apoptosis [52–54]. SETD8 overexpression was reported in various solid cancers, such as bladder cancer, non-small cell lung and small cell lung carcinomas, hepatocellular carcinoma and pancreatic cancer [55]. SETD8 is also involved in hematologic cancers. It is overexpressed in chronic myelogenous leukemia (CML) [55], and up-regulated in high stage chronic lymphocytic leukemia (CLL) [56]. Single-nucleotide polymorphisms (SNPs) at the miRNA binding site in the 3’-untranslated region of *SETD8* are associated with risk of pediatric acute lymphoblastic leukemia (ALL) [57] and have a prognostic impact in NHL [58]. Potent SETD8 inhibitors (SETD8i) have been developed with effects in human leukemic cell lines [53, 59].

We also identified other epigenetic genes with significant relevance in DLBCL. *HDAC2* was found to have adverse prognostic value in DLBCL [60]. *HDAC2* not only facilitates lymphomagenesis, but is also required for lymphoma maintenance [61]. Moreover, HDAC inhibitors (HDACi) appear to be promising therapeutic agents in patients with DLBCL because they can restore sensitization of DLBCL cells to CHOP [62, 63]. High *PRMT5* gene expression has been associated with poor prognosis in DLBCL cohorts. *PRMT5* is a key modulator of lymphomagenesis [64] . This suggests that PRMT5 inhibition could be a novel therapeutic approach for B-cell lymphoma and PRMT5 inhibitors (PRMT5i) are currently in pre-clinical development [65]. BRD and BET proteins are epigenetic “readers” of histone post-translational modifications involved in chromatin remodeling and transcriptional regulation. BET and BRD inhibitors (BETi and BRDi) have shown efficiency in refractory hematologic malignancies and more specifically in DLBCL [66]. In the present study, among the *BET* and *BRD* genes explored for their prognostic value in DLBCL, *BRD1* expression was associated with poor outcome. BRD1 localizes to the nucleus and can interact with DNA and histones. Alternative splicing results in multiple transcript variants and some variants are involved in malignant mesothelioma [67]. Moreover, BRD1 inhibitors (BRD1i) could represent an interesting therapeutic option in DLBCL [68]. However, expression changes of these epigenetic genes could be transient and the levels would be restored following the drug withdrawal.

Interestingly, the GSEA analysis highlighted a significant enrichment of genes encoding for HDAC class I and mTOR (mechanistic target of rapamycin) pathways and APC (adenomatosis polyposis coli) and HDAC7 targets (Supplementary Figure 3 and Supplementary Tables 2–5) in patients in the EpiScore high-risk group. HDACi lead to cell arrest, induce apoptosis, can have an anti-angiogenic and inhibitory effect on the occurrence of metastasis in solid cancers, can contribute to inhibition of various proteins involved in DNA repair and may increase immunogenicity of neoplastic cells [69]. HDACi also sensitizes cycling cells to irradiation and DNA-targeting drugs [70, 71]. More precisely, HDACi acts on chromatin structure during DSB repair process and downregulates the activity of DNA repair machinery [72, 73]. In AML HDACi induce cell differentiation and apoptosis through accumulation of DNA damage and inhibition of DNA repair [74]. Vorinostat, and panobinostat, inhibitors of HDAC class I and II, are effective in patients with hematologic malignancies in phase 1 and phase 2 clinical trials [75–77] and vorinostat selectively down-regulates HDAC7 [78]. These data further support that targeting HDAC could have therapeutic interest in high-risk DLBCL patients identified using EpiScore. In line with these results, DLBCL cell lines overexpressing 2 out of the 3 genes of the EpiScore are significantly more sensitive to SAHA than SUDHL5 characterized by low expression of the 3 genes. A clinical validation of the EpiScore interest to identify DLBCL patients that could benefit from HDACi treatment will be important. The mTOR pathway may be activated in lymphoma cells [79, 80] and its activation in patients with DLBCL is associated with unfavorable prognosis, poor response to treatment and decreased survival time [81]. mTOR inhibitors combined with R-CHOP have shown promising results in untreated patients with DLBCL in a phase 1 clinical trial [82].

Finally, although we demonstrated that EpiScore robustly segregates patients with DLBCL in three prognostic groups, it is currently impractical to perform microarray analysis on all patients with DLBCL in the routine practice. Thus, we focused on SETD8, DOT1L and DNMT3A protein expression in DLBCL. We found that DNMT3A protein overexpression correlates with OS and EFS. DNMT3A protein overexpression, detected with immunohistochemistry, has been previously linked to pejorative prognosis in solid cancers, such as retinoblastoma [83], breast carcinoma [84] or gastroenteropancreatic neuroendocrine tumours [85]. DOT1L protein expression, a trend was identified for an association between no detectable DOT1L expression and a better EFS in DLBCL samples. Future studies should be conducted to validate the prognostic value of DOT1L protein expression in larger cohort of patients. In the present study, anti-SETD8 antibodies were not specific, and no study has been published on SETD8 protein expression in cancer specimens by immunohistochemistry. More efficient antibodies should be developed to evaluate SETD8 expression in formalin-fixed, paraffin-embedded tissues.

In conclusion, given the molecular heterogeneity of patients with DLBCL, we designed the EpiScore to identify high-risk patients who could benefit from aggressive treatments and new epigenetic therapies. We also show that DNMT3A overexpression, which can be easily evaluated in the routine practice, is a new potential prognostic factor that could be used to identify high-risk patients with DLBCL.

## MATERIALS AND METHODS

### Gene expression data of patients with DLBCL

Gene expression microarray data from two independent cohorts of patients diagnosed with DLBCL were used. The first cohort (*n* = 414 patients; Lenz cohort) [21] was further divided in two cohorts, according to the patients’ treatment. The first one, used as training cohort, included 233 patients treated with R-CHOP, whereas the second one, used as validation cohort, comprised 181 patients treated with CHOP. A third cohort (69 patients treated with R-CHOP; Melnick cohort) also was used as validation cohort [86]. The pre-treatment clinical characteristics of the Lenz and Melnick cohorts were previously published by G. Lenz and R. Shaknovich’s groups, respectively [21, 86]. Affymetrix gene expression data (obtained using Affymetrix HG-U133 plus 2.0 microarrays) are publicly available *via* the online Gene Expression Omnibus (http://www.ncbi.nlm.nih.gov/geo/) under the accession numbers GSE10846 and GSE23501. We also used GSE56315 data to compare gene expression profiles between DLBCL and normal centrocyte and centroblast samples [24]. They were analyzed with Microarray Suite version 5.0 (MAS 5.0), using Affymetrix default analysis settings and global scaling as normalization method. The trimmed mean target intensity of each array was arbitrarily set to 500.

### Gene expression profiling and statistical analyses

The statistical significance of OS differences between groups was calculated using the log-rank test. Multivariate analysis was performed using the Cox proportional hazards model and Genomicscape (http://genomicscape.com) [87]. Survival curves were plotted using the Kaplan-Meier method. All analyses were done with R.2.10.1 and Bioconductor version 2.5.

### Selection of prognostic genes in the training set

Probe sets were selected for prognostic significance using the Maxstat R function and Benjamini Hochberg multiple testing correction [22, 23] and the expression data from the two Lenz cohorts (*n* = 233 patients and *n* = 181 patients ) [21].

### Building the epigenetic gene expression-based risk score (EpiScore)

To gather prognostic information of the prognostic genes, the EpiScore was built as the sum of the beta coefficients weighted by ± 1, according to the patient signal above or below the probe set Maxstat cut-off as previously described [22].

### Validation in the independent cohort of patients

EpiScore was individually calculated for each patient and patients were grouped according to the prognostic model and cut-offs from the training cohort. The prognostic value of this scoring was evaluated using the log-rank test and Cox models.

### Gene set enrichment analysis (GSEA)

We compared the gene expression levels in high risk EpiScore versus low risk EpiScore patients with DLBCL and identified the genes with significant different expression using GSEA. GSEA was carried out by computing the overlaps with canonical pathways and gene ontology gene sets obtained from the Broad Institute (Cambridge, USA) [88].

### immunohistochemistry

Tumor samples from patients with DLBCL from the Department of Pathology of the Montpellier University Hospital were selected for immunohistochemical analysis. The diagnosis of DLBCL was based on the World Health Organization (WHO) 2008 classification of tumors of hematopoietic and lymphoid tissues [1]. All cases were systematically reviewed by two expert pathologists (VS, VC). Tissue microarrays (TMA) containing three representative 0.6-mm cores of routinely processed tissues from patients with DLBCL with available FFPE tissue blocks were prepared (Beecher Instruments, Silver Spring, MD). Only patients with a large tumor sample were selected for TMA. The quality of each tissue core was evaluated based on its morphology, using hematoxylin and eosin staining, and the percentage of CD20+ tumor cells. Only tissue cores with more than 50% CD20+ tumor cells were retained for immunohistochemical analysis. In parallel, five FFPE non-neoplastic samples (two reactive lymph node and three tonsil specimens) were included and used as controls. Three µm-thick tissue sections from paraffin blocks were immunostained on a Ventana Benchmark XT autostainer (Ventana Tucson, AZ, USA). The following antibodies were used after the appropriate antigen retrieval procedure according to the manufacturer’s instructions: anti-DOT1L (clone NB100-40845, Novus Biologicals, Ltd., Cambridge, UK, 1:50) [89], -DNMT3A (clone H-295, Santa Cruz Biotechnology, 1:200), -SETD8 (clone 43AT551.86, LSBio, 1:800), -MYC (clone EP 121, Epitomics, Burlingame, CA, USA 1:100), -P53 (clone DO7, Ventana, PREP Kit Ventana), -KI67 (clone 30-9, Ventana, PREP Kit Ventana) and -BCL2 (clone 124 Dako, 1:100). The study was approved by the ethic committee of Montpellier and patients provided a written informed consent (DC-2010-1185 and DC-2013-2027). For protein expression evaluation, slides were digitized using an iScan Coreo scanner (Ventana, Roche, France) to generate images. The Ventana image analysis algorithm, which is integrated in the Ventana Virtuoso image and workflow management software, was used for detection and semi-quantitative measurement of each protein (Ventana, Roche, France). Immunostaining results (i.e., percentage of positive cells) were evaluated by two pathologists. P53 and MYC expression were considered positive if nuclear staining was respectively observed in 10% or more and in 40% or more of tumor cells [90–94]. BCL2 expression was scored as positive if 50% or more of tumor cells showed cytoplasmic staining [90]. High KI67 expression was considered when more than 80% of tumor cells showed nuclear staining [95]. Clinical and follow-up data concerning performance status, number of extranodal sites, serum lactate dehydrogenase (LDH) level, international prognostic index, response to treatment and survival were available for all patients. Patients were uniformly treated at the same institution with standard regimens, according to their IPI scores and age, and completed their planned treatment. Twenty-seven patients were treated with R-CHOP, one with rituximab, cyclophosphamide, vincristine (oncovin™) and prednisone (R-COP), two with dexamethasone, high dose cytarabine, cisplatin (DHAP) and carmustine, etoposide, cytarabine, melphalan (BEAM), one with rituximab, dexamethasone, doxorubicin, cytarabine, carboplatin (R-DHAC) and BEAM. The patients’ outcome was evaluated according to standard international criteria [96].

### Human DLBCL cell lines

Human DLBCL cell lines (DB, RI1, NUDUL1 and SUDHL5) were from DSMZ (Germany). Cell pellet were fixed in formalin and then embedded on paraffin. Three µm-thick tissue sections from paraffin blocks were immunostained on a Ventana Benchmark XT with anti-DOT1L, -DNMT3A, -SETD8 antibodies.

## SUPPLEMENTARY MATERIALS FIGURES AND TABLES




