# Supplementary material for: An epigenetic regulator-related score (EpiScore) predicts survival in patients with diffuse large B cell lymphoma and identifies patients who may benefit from epigenetic therapy
Source: Oncotarget. 2018 Apr 10;9(27):19079–99. doi: 10.18632/oncotarget.24901 (PMC5922379; doi:10.18632/oncotarget.24901)
Supplement: Supplementary file 1 [file oncotarget-09-19079-s001.pdf]

An epigenetic regulator-related score (EpiScore) predicts survival in patients with diffuse large B cell lymphoma and identifies patients who may benefit from epigenetic therapy

SUPPLEMENTARY MATERIALS

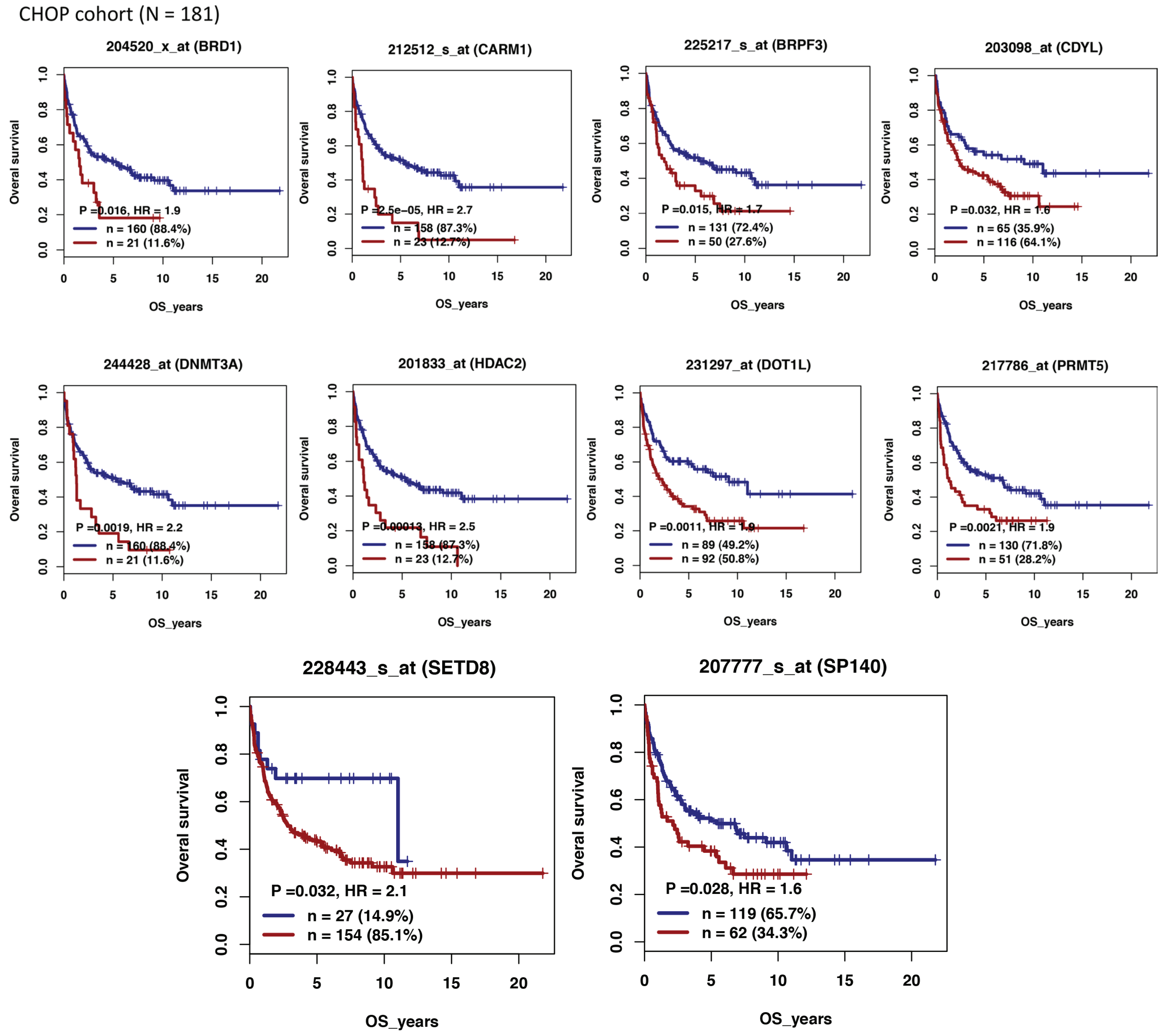

Supplementary Figure 1: Epigenetic genes with prognostic value in patients with DLBCL. The prognostic value of the ten genes was validated in an independent cohort (Lenz CHOP cohort n = 181). For a given gene, a prognostic expression cut-off was calculated using the Maxstat algorithm, as described in Materials and Methods, to split patients in two groups (high and low risk) according to their overall survival (OS). *BRD1*: bromodomain containing 1; *CARM1*: coactivator associated arginine methyltransferase 1; *BRPF3*: bromodomain and PHD finger containing 3; *CDYL:* chromodomain Y like; *DNMT3A:* DNA cytosine-5-methyltransferase 3 alpha; *DOT1L*: DOT1-like histone H3K79 methyltransferase; HDAC2: histone deacetylase 2; *PRMT5:* protein arginine methyltransferase 5; *SETD8*: also known as KMT5 lysine (K)-specific methyltransferase 5A; *SP140*: SP140 nuclear body protein.

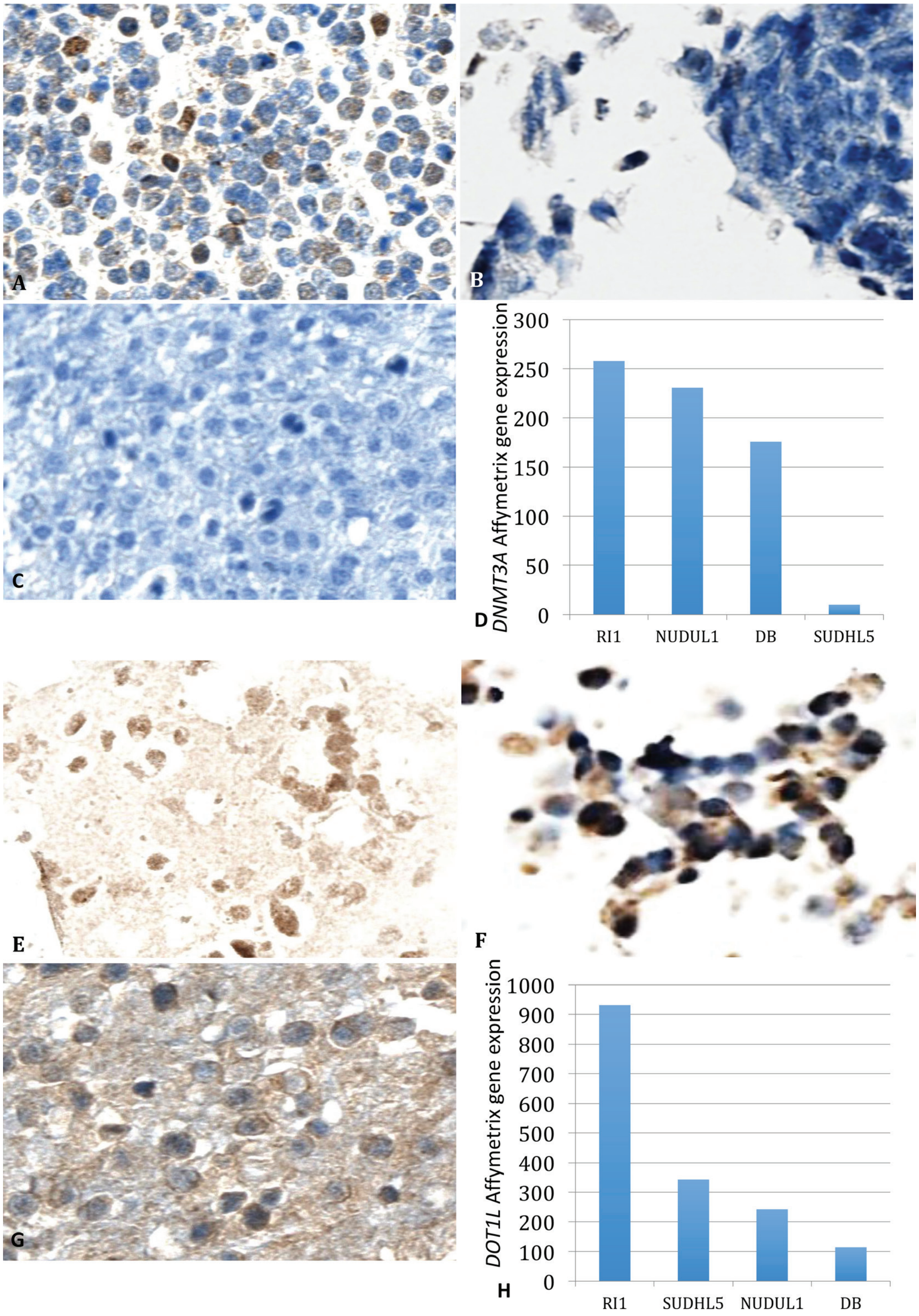

Supplementary Figure 2: DNMT3A and DOT1Lprotein expression in DLBCL cell lines. (**A**) Immunohistochemical staining for DNMT3A in RI1 cells (known to have high *DNMT3A* gene expression). 35% of cells show DNMT3A nuclear expression. (**B**) Immunohistochemical staining for DNMT3A in DB cells (known to have high *DNMT3A* gene expression). 5% of cells show DNMT3A nuclear expression. (**C**) Immunohistochemical staining for DNMT3A in SUDHL5 cells (known to have low *DNMT3A* gene expression). No DNMT3A expression could be detected. (**D**) *DNMT3A* Affymetrix gene expression in DLBCL cells. (**E**) Immunohistochemical staining for DOT1L in RI1 cells (known to have high *DOT1L* gene expression). 80% of cells show DOT1L nuclear expression. (**F**) Immunohistochemical staining for DOT1L in SUDHL5 cells (known to have high *DOT1L* gene expression). 40% of cells show DOT1L nuclear expression. (**G**) Immunohistochemical staining for DOT1L in DB cells (known to have low *DOT1L* gene expression). No DOT1L expression could be detected. (**H**) *DOT1L* Affymetrix gene expression in DLBCL cells.

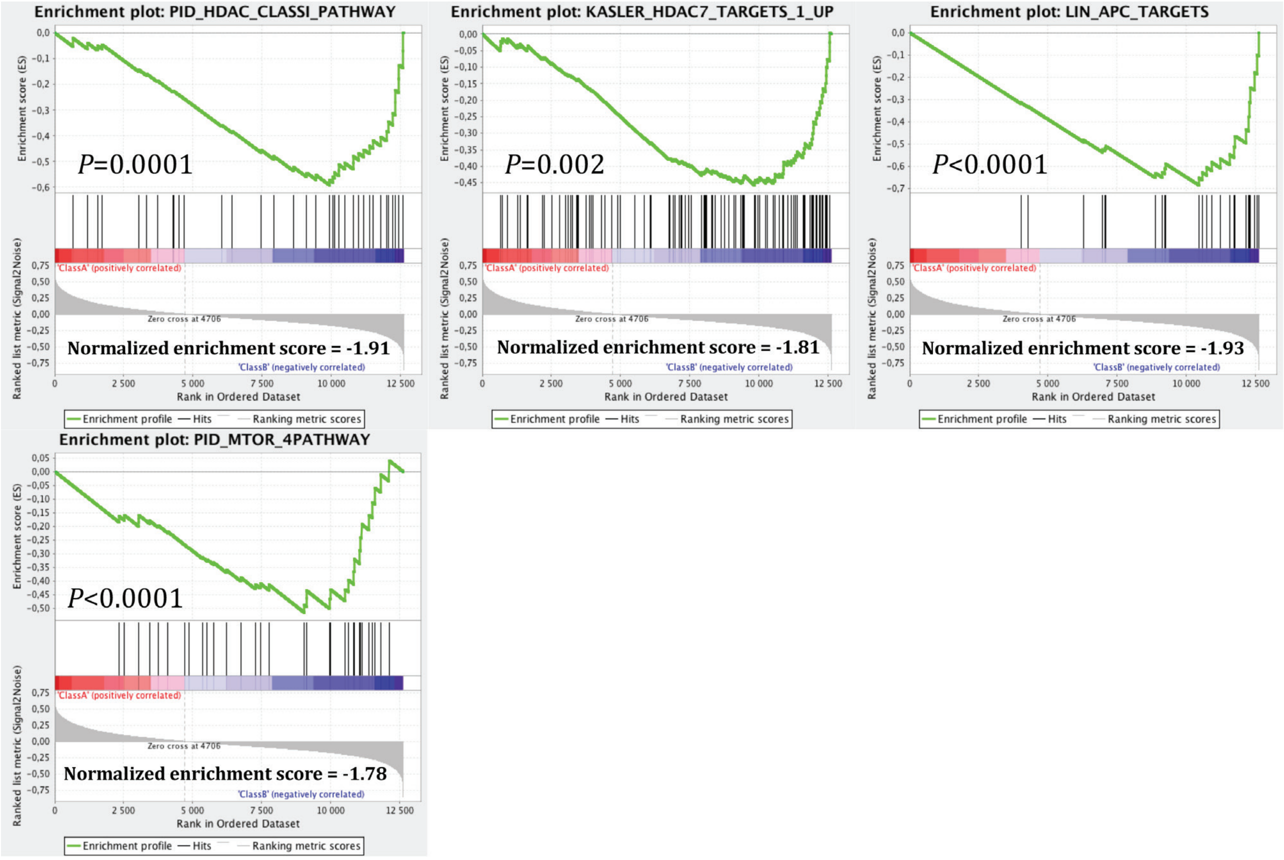

Supplementary Figure 3: Gene set enrichment (GSEA) analysis of the gene expression profiles of DLBCL samples from patients at high or low risk according to EpiScore. The EpiScore high risk group showed significant enrichment in genes related to the Class I HDAC pathway, HDAC7 targets, APC targets and mTOR pathway. HDAC: histone deacetylase; mTOR mechanistic target of rapamycin; APC: adenomatosis polyposis coli.

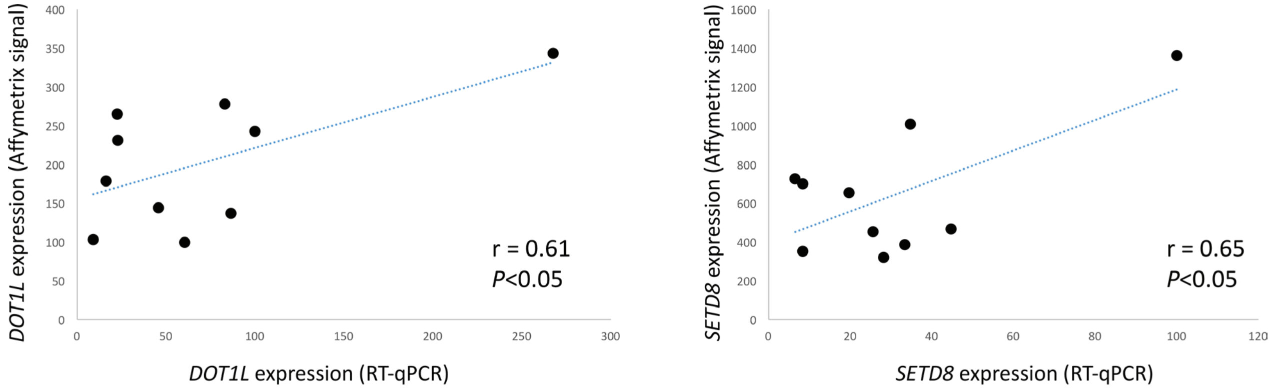

Supplementary Figure 4: Validation of *SETD8* and *DOT1L* Affymetrix data. *SETD8* and *DOT1L* gene expression was assayed by RT-qPCR and the correlation between Affymetrix and RT-qPCR was determined using a Spearman's test in 10 DLBCL cell lines.

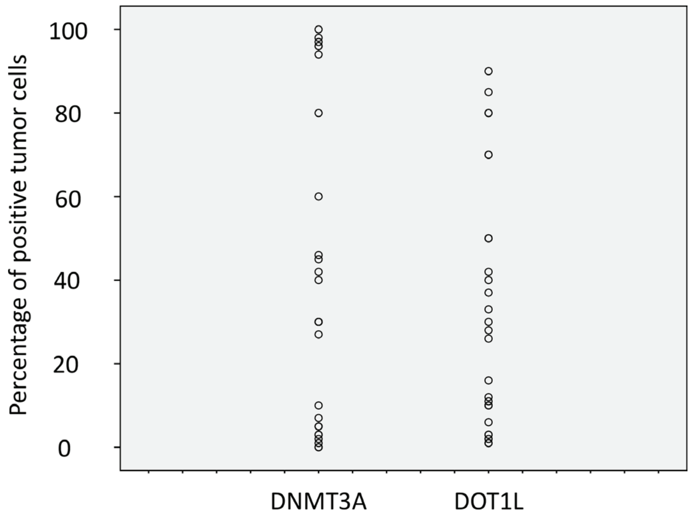

Supplementary Figure 5: Distribution of DNMT3A and DOT1L protein expression in TMA samples of 31 patients with DLBCL.

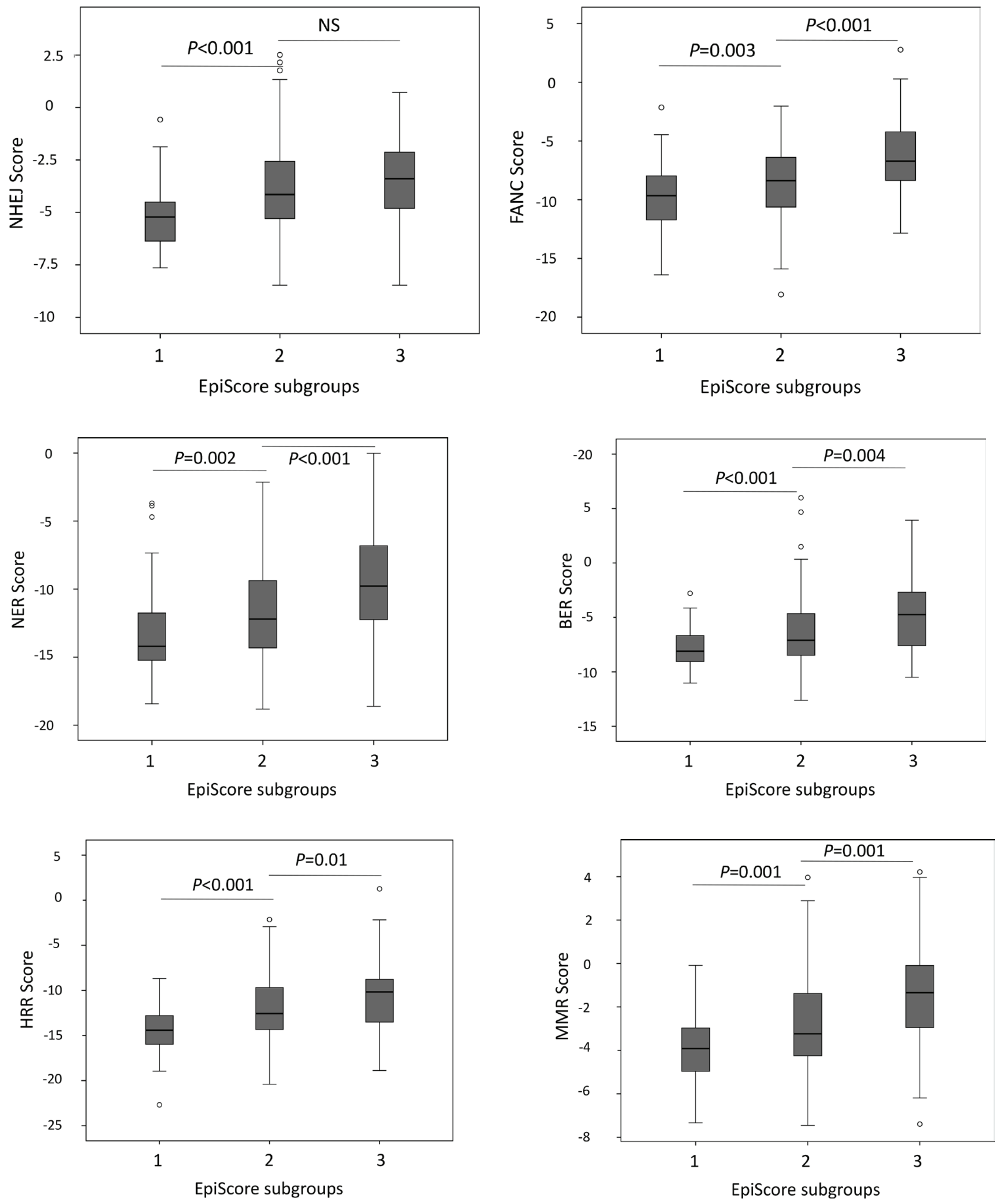

Supplementary Figure 6: NHEJ, FANC, NER, BER, HRR and MMR DNA repair score values in the three EpiScore subgroups of DLBCL patients using the R-CHOP Lenz cohort.

Supplementary Table 1: List of epigenetic genes under study. See Supplementary_Table_1

Supplementary Table 2: Gene set enrichment analysis revealed a significant over-representation of the gene set PID_HDAC_CLASSI_PATHWAY in patients with diffuse large B-cell lymphoma in the EpiScore high risk group compared with patients in the low risk group (*p* = 0.0001)

GENE SYMBOL	GENE_TITLE	RANK METRIC SCORE	RUNNING ES	CORE ENRICHMENT
NCOR1	nuclear receptor co-repressor 1	–0.172743976	–0.5718318	Yes
TNFRSF1A	tumor necrosis factor receptor superfamily, member 1A	–0.178016603	–0.5600956	Yes
RBBP4	retinoblastoma binding protein 4	–0.181025356	–0.5434029	Yes
SIRT3	sirtuin (silent mating type information regulation 2 homolog) 3 (S. cerevisiae)	–0.188710287	–0.534076	Yes
NFKBIA	nuclear factor of kappa light polypeptide gene enhancer in B-cells inhibitor, alpha	–0.189279333	–0.5130078	Yes
PPARG	peroxisome proliferative activated receptor, gamma	–0.201698065	–0.5066175	Yes
SIN3A	SIN3 homolog A, transcription regulator (yeast)	–0.218119219	–0.50451154	Yes
HDAC11	histone deacetylase 11	–0.218844518	–0.47984487	Yes
RELA	v-rel reticuloendotheliosis viral oncogene homolog A, nuclear factor of kappa light polypeptide gene enhancer in B-cells 3, p65 (avian)	–0.231569454	–0.4680731	Yes
NFKB1	nuclear factor of kappa light polypeptide gene enhancer in B-cells 1 (p105)	–0.244279623	–0.45386952	Yes
PRMT5	protein arginine methyltransferase 5	–0.260750681	–0.43949693	Yes
YY1	YY1 transcription factor	–0.274263889	–0.41854864	Yes
SIRT6	sirtuin (silent mating type information regulation 2 homolog) 6 (S. cerevisiae)	–0.308375329	–0.40459195	Yes
SIRT7	sirtuin (silent mating type information regulation 2 homolog) 7 (S. cerevisiae)	–0.337804168	–0.38101652	Yes
SIN3B	SIN3 homolog B, transcription regulator (yeast)	–0.351141065	–0.34548518	Yes
MAX	MYC associated factor X	–0.392509639	–0.3134008	Yes
GATAD2B	GATA zinc finger domain containing 2B	–0.416231871	–0.27069378	Yes
SIRT2	sirtuin (silent mating type information regulation 2 homolog) 2 (S. cerevisiae)	–0.416265517	–0.22226444	Yes
ZFPM1	zinc finger protein, multitype 1	–0.461861134	–0.17821988	Yes
MBD3	methyl-CpG binding domain protein 3	–0.474458337	–0.12476754	Yes
SMG5	Smg-5 homolog, nonsense mediated mRNA decay factor (C. elegans)	–0.579334795	–0.06840598	Yes
GATAD2A	GATA zinc finger domain containing 2A	–0.609816194	0.001667847	Yes

Supplementary Table 3: Gene set enrichment analysis revealed a significant over-representation of the gene set KASLER_HDAC7_TARGET_1_UP in patients with diffuse large B-cell lymphoma in the EpiScore high risk group compared with patients in the EpiScore low risk group (*p* = 0.002)

GENE SYMBOL	GENE_TITLE	RANK METRIC SCORE	RUNNING ES	CORE ENRICHMENT
SLCO3A1	solute carrier organic anion transporter family, member 3A1	–0.168471649	–0.44797662	Yes
CNNM3	cyclin M3	–0.169821471	–0.44103208	Yes
HIP1R	huntingtin interacting protein 1 related	–0.174069211	–0.43904155	Yes
RASAL1	RAS protein activator like 1 (GAP1 like)	–0.177079484	–0.43489024	Yes
GM2A	GM2 ganglioside activator	–0.187352479	–0.44110775	Yes
PCYT1A	phosphate cytidylyltransferase 1, choline, alpha	–0.190964267	–0.43595347	Yes
ARHGEF2	rho/rac guanine nucleotide exchange factor (GEF) 2	–0.202950865	–0.44139302	Yes
SYVN1	synovial apoptosis inhibitor 1, synoviolin	–0.20625262	–0.4343605	Yes
AGPAT6	1-acylglycerol-3-phosphate O-acyltransferase 6 (lysophosphatidic acid acyltransferase, zeta)	–0.206799164	–0.42378637	Yes
LATS2	LATS, large tumor suppressor, homolog 2 (Drosophila)	–0.214373112	–0.42349005	Yes
ASB6	ankyrin repeat and SOCS box-containing 6	–0.221519142	–0.4211248	Yes
U2AF2	U2 small nuclear RNA auxiliary factor 2	–0.223186374	–0.4108466	Yes
ANKRD9	ankyrin repeat domain 9	–0.223581254	–0.39895052	Yes
LRP10	low density lipoprotein receptor-related protein 10	–0.224740312	–0.3878686	Yes
MARCH3	membrane-associated ring finger (C3HC4) 3	–0.234317049	–0.38527843	Yes
ZBTB7B	zinc finger and BTB domain containing 7B	–0.244465098	–0.38340712	Yes
AKAP12	A kinase (PRKA) anchor protein (gravin) 12	–0.251035571	–0.3759869	Yes
MIER2	mesoderm induction early response 1, family member 2	–0.25761649	–0.36780578	Yes
RARA	retinoic acid receptor, alpha	–0.260573357	–0.35658896	Yes
USP19	ubiquitin specific peptidase 19	–0.282144874	–0.35807252	Yes
ARHGAP27	Rho GTPase activating protein 27	–0.287364841	–0.34673914	Yes
FBXL6	F-box and leucine-rich repeat protein 6	–0.289139628	–0.33171675	Yes
GRB2	growth factor receptor-bound protein 2	–0.316613227	–0.32938945	Yes
NHEJ1	nonhomologous end-joining factor 1	–0.325953513	–0.31665227	Yes
TMEM57	transmembrane protein 57	–0.329488128	–0.30052838	Yes
PEX6	peroxisomal biogenesis factor 6	–0.332910031	–0.28373748	Yes
SHKBP1	SH3KBP1 binding protein 1	–0.334608883	–0.26597524	Yes
CYFIP2	cytoplasmic FMR1 interacting protein 2	–0.354989737	–0.25507307	Yes
TRIM35	tripartite motif-containing 35	–0.356456369	–0.2361094	Yes
TMUB1	transmembrane and ubiquitin-like domain containing 1	–0.380855769	–0.22338577	Yes
DGKZ	diacylglycerol kinase, zeta 104kDa	–0.38845098	–0.20409922	Yes
RAB1B	RAB1B, member RAS oncogene family	–0.405480564	–0.18611082	Yes
TDP1	tyrosyl-DNA phosphodiesterase 1	–0.415417284	–0.16550097	Yes
AP1M1	adaptor-related protein complex 1, mu 1 subunit	–0.456527263	–0.14989297	Yes
SBF1	SET binding factor 1	–0.45907256	–0.12488723	Yes
IKZF1	IKAROS family zinc finger 1 (Ikaros)	–0.472208709	–0.10059566	Yes
POLDIP2	polymerase (DNA-directed), delta interacting protein 2	–0.481293112	–0.07612376	Yes
DNMT3A	DNA (cytosine-5-)-methyltransferase 3 alpha	–0.526865363	–0.053056374	Yes
MRPS2	mitochondrial ribosomal protein S2	–0.5272246	–0.02406341	Yes
SLC35A4	solute carrier family 35, member A4	–0.546430111	0.004229913	Yes

Supplementary Table 4: Gene set enrichment analysis revealed a significant overrepresentation of the gene set LIN_APC_TARGETS in patients with diffuse large B-cell lymphoma in the EpiScore high risk group compared with patients in the EpiScore low risk group (*p* < 0.0001)

GENE SYMBOL	GENE_TITLE	RANK METRIC SCORE	RUNNING ES	CORE ENRICHMENT
GNA12	guanine nucleotide binding protein (G protein) alpha 12	–0.199666321	–0.65353423	Yes
CAPN1	calpain 1, (mu/I) large subunit	–0.205778122	–0.6285446	Yes
PCDH9	protocadherin 9	–0.215122998	–0.60710055	Yes
ETS1	v-ets erythroblastosis virus E26 oncogene homolog 1 (avian)	–0.225786105	–0.5837508	Yes
NELF	nasal embryonic LHRH factor	–0.248253271	–0.5675869	Yes
AES	amino-terminal enhancer of split	–0.279112846	–0.54842937	Yes
SYK	spleen tyrosine kinase	–0.300042331	–0.5122788	Yes
MLLT6	myeloid/lymphoid or mixed-lineage leukemia (trithorax homolog, Drosophila); translocated to, 6	–0.304607153	–0.46522722	Yes
ZBTB7A	zinc finger and BTB domain containing 7A	–0.372826874	–0.4354747	Yes
SF3A1	splicing factor 3a, subunit 1, 120kDa	–0.403069049	–0.37768486	Yes
RAB1B	RAB1B, member RAS oncogene family	–0.405480564	–0.31172648	Yes
SEC61A1	Sec61 alpha 1 subunit (S. cerevisiae)	–0.419011623	–0.24632739	Yes
GLTSCR2	glioma tumor suppressor candidate region gene 2	–0.475793689	–0.17963648	Yes
ARFGAP1	ADP-ribosylation factor GTPase activating protein 1	–0.529709816	–0.10054248	Yes
TELO2	TEL2, telomere maintenance 2, homolog (S. cerevisiae)	–0.647063673	6.34E-04	Yes

Supplementary Table 5: Gene set enrichment analysis revealed a significant over-representation of the gene set PID_ MTOR_4PATHWAY in patients diffuse large B-cell lymphoma in the EpiScore high risk group compared with patients in the EpiScore low risk group (*p* < 0.0001)

GENE SYMBOL	GENE_TITLE	RANK METRIC SCORE	RUNNING ES	CORE ENRICHMENT
ULK1	unc-51-like kinase 1 (C. elegans)	–0.136622399	–0.48699063	Yes
MAPK1	mitogen-activated protein kinase 1	–0.13936533	–0.46379092	Yes
AKT1S1	AKT1 substrate 1 (proline-rich)	–0.139520481	–0.4348432	Yes
EIF4E	eukaryotic translation initiation factor 4E	–0.173422158	–0.464156	Yes
PRR5	proline rich 5 (renal)	–0.175257221	–0.43021387	Yes
RRAGC	Ras-related GTP binding C	–0.203071654	–0.43039927	Yes
YWHAG	tyrosine 3-monooxygenase/tryptophan 5-monooxygenase activation protein, gamma polypeptide	–0.20958437	–0.39564657	Yes
CYCS	cytochrome c, somatic	–0.220453128	–0.36561227	Yes
RRN3	RRN3 RNA polymerase I transcription factor homolog (S. cerevisiae)	–0.221275926	–0.32040274	Yes
PML	promyelocytic leukemia	–0.235946521	–0.28745374	Yes
MAP2K2	mitogen-activated protein kinase kinase 2	–0.238051504	–0.2403322	Yes
RRAGD	Ras-related GTP binding D	–0.240720108	–0.19273332	Yes
EEF2K	eukaryotic elongation factor-2 kinase	–0.262093306	–0.15925145	Yes
YY1	YY1 transcription factor	–0.274263889	–0.111878686	Yes
PDPK1	3-phosphoinositide dependent protein kinase-1	–0.284686118	–0.06018828	Yes
MAPKAP1	mitogen-activated protein kinase associated protein 1	–0.309892058	–0.010701549	Yes
YWHAB	tyrosine 3-monooxygenase/tryptophan 5-monooxygenase activation protein, beta polypeptide	–0.36256519	0.040644582	Yes
