# Supplementary material for: An epigenetic regulator-related score (EpiScore) predicts survival in patients with diffuse large B cell lymphoma and identifies patients who may benefit from epigenetic therapy
Source: Oncotarget. 2018 Apr 10;9(27):19079–99. doi: 10.18632/oncotarget.24901 (PMC5922379; doi:10.18632/oncotarget.24901)
Supplement: Supplementary file 2 [file oncotarget-09-19079-s002.docx]

| **Genes encoding epigenetic regulators** |
| --- |
|  |
| **DNA methyltransferases (DNMTs; n = 4)** |
| *DNMT1* |
| *TRDMT1* |
| *DNMT3A* |
| *DNMT3B* |
| **Methyl-binding proteins (MBDs; n = 5)** |
| *MBD1* |
| *MBD2* |
| *MBD3* |
| *MBD4* |
| *MECP2* |
| **Histone acetyltransferases (HATs; n = 19)** |
| P300/CBP |
| *CREBBP* |
| *EP300* |
| HAT1 |
| *HAT1* |
| MYST |
| *MYST1* |
| *MYST2* |
| *MYST3* |
| *MYST4* |
| *HTATIP* |
| GCN5 |
| *PCAF* |
| *GCN5L2* |
| p160 |
| *NCOA1* |
| *NCOA3* |
| Others |
| *SRCAP* |
| *TAF1* |
| *TAF1L* |
| *GTF3C4* |
| *ATF2* |
| *CDY1* |
| *CDYL* |
| **Histone deacetylases (HDACs; n = 13)** |
| Class I |
| *HDAC1* |
| *HDAC2* |
| *HDAC3* |
| *HDAC8* |
| Class II |
| *HDAC4* |
| *HDAC5* |
| *HDAC6* |
| *HDAC7A* |
| *HDAC9* |
| *HDAC10* |
| Class III |
| *SIRT1* |
| *SIRT2* |
| Class IV |
| *HDAC11* |
| **Histone methylatransferases (HMTs; n = 41)** |
| Arginine HMTs |
| *PRMT1* |
| *PRMT5* |
| *PRMT7* |
| *PRMT8* |
| *CARM1* |
| Lysine HMTs |
| SUV39 |
| *EHMT1* |
| *EHMT2* |
| *SUV39H1* |
| *SUV39H2* |
| *SUV420H2* |
| *SETDB1* |
| *SETDB2* |
| SET1 |
| *SETD7* |
| *SETD8* |
| *MLL* |
| *MLL2* |
| *MLL3* |
| *MLL4* |
| *EZH1* |
| *EZH2* |
| SET2 |
| *NSD1* |
| *WHSC1* |
| *WHSC1L1* |
| *ASH1L* |
| RIZ |
| *PRDM1* |
| *PRDM2* |
| *PRDM3* |
| *PRDM4* |
| *PRDM5* |
| *PRDM6* |
| *PRDM7* |
| *PRDM8* |
| *PRDM9* |
| *PRDM10* |
| *PRDM11* |
| *PRDM12* |
| *PRDM13* |
| *PRDM14* |
| *PRDM15* |
| *PRDM16* |
| Other |
| *SMYD3* |
| **Histone demethylases (n = 8)** |
| *PADI4* |
| *AOF2* |
| *JMJD2A* |
| *JMJD2C* |
| *JARID1B* |
| *JMJD1A* |
| *FBXL10* |
| *FBXL11* |
| **Bromodomain (BRD) and Extra-Terminal motif (BET) proteins (n=40)** |
| ASH1L |
| ATAD2 |
| BAZ1A |
| BAZ1B |
| BAZ2A |
| BAZ2B |
| BRD1 |
| BRD2 |
| BRD3 |
| BRD4 |
| BRD7 |
| BRD8B |
| BRD9 |
| BRDT |
| BRPF1 |
| BRPF3 |
| BRWD3 |
| CECR2 |
| CREBBP |
| EP300 |
| FALZ |
| GCN5L2 |
| MLL |
| PB1 |
| PCAF |
| PHIP |
| PRKCBP1 |
| SMARCA2 |
| SMARCA4 |
| SP100 |
| SP110 |
| SP140 |
| TAF1 |
| TAF1L |
| TRIM24 |
| TRIM28 |
| TRIM33 |
| TRIM66 |
| WDR9 |
| ZMYND11 |

**TABLE S1: List of epigenetic genes under study.**
